# Public attitudes towards the use of automatic facial recognition technology in criminal justice systems around the world

**DOI:** 10.1371/journal.pone.0258241

**Published:** 2021-10-13

**Authors:** Kay L. Ritchie, Charlotte Cartledge, Bethany Growns, An Yan, Yuqing Wang, Kun Guo, Robin S. S. Kramer, Gary Edmond, Kristy A. Martire, Mehera San Roque, David White

**Affiliations:** 1 School of Psychology, University of Lincoln, Lincoln, Lincolnshire, United Kingdom; 2 School of Psychology, University of New South Wales, Sydney, New South Wales, Australia; 3 School of Social and Behavioural Sciences, Arizona State University, Tempe, Arizona, United States of America; 4 State Key Laboratory of Cognitive Neuroscience and Learning and IDG/McGovern Institute for Brain Research, Beijing Normal University, Beijing, China; 5 Faculty of Law and Justice, University of New South Wales, Sydney, New South Wales, Australia; Imam Abdulrahman Bin Faisal University, SAUDI ARABIA

## Abstract

Automatic facial recognition technology (AFR) is increasingly used in criminal justice systems around the world, yet to date there has not been an international survey of public attitudes toward its use. In Study 1, we ran focus groups in the UK, Australia and China (countries at different stages of adopting AFR) and in Study 2 we collected data from over 3,000 participants in the UK, Australia and the USA using a questionnaire investigating attitudes towards AFR use in criminal justice systems. Our results showed that although overall participants were aligned in their attitudes and reasoning behind them, there were some key differences across countries. People in the USA were more accepting of tracking citizens, more accepting of private companies’ use of AFR, and less trusting of the police using AFR than people in the UK and Australia. Our results showed that support for the use of AFR depends greatly on what the technology is used for and who it is used by. We recommend vendors and users do more to explain AFR use, including details around accuracy and data protection. We also recommend that governments should set legal boundaries around the use of AFR in investigative and criminal justice settings.

## Introduction

Biometrics refers to the characteristics of a person which can be used to identify them [1]. The most common forms of biometrics used in law enforcement and other security settings are fingerprints, iris, voice, DNA and face. Over the past decade or more, the use of biometric has grown rapidly, particularly in investigative and criminal justice settings, often in response to terrorism [2,3]. Facial recognition technology is an increasingly common form of biometrics in use in many different areas of our lives–from unlocking smart devices, to crossing borders, and increasingly in security and policing settings.

Automatic facial recognition (AFR) technology is based on algorithms that perform a series of functions, including detecting a face, creating a digital representation–or ‘template’– of the face, and comparing this representation against other images to determine the degree of similarity between them. Here we focus solely on AFR technology which performs two main functions: verification and identification. Verification is an identity confirmation based on a one-to-one comparison of a single stored image, for example on a passport, to another single face image, for example an image taken by an automated border control gate. Identification is a one-to-many (1:N) search of a database, for example a criminal watchlist, to find a match to the target image, which could for example be a CCTV image of someone committing a crime. Verification and identification might be performed by a person or an algorithm or by combinations of one or more persons and algorithms. A detailed discussion of the operational uses of these types of algorithms is provided elsewhere [4,5].

In this paper we report a study aimed at understanding public opinion towards use of this technology in society, with a focus on how the technology is used in the criminal justice system. Before describing our study, we provide background on: 1) AFR algorithm accuracy; 2) AFR algorithm bias; 3) use and governance of AFR; 4) public opinion of AFR; 5) the current study.

### Algorithm accuracy

In recent years, there has been a rapid improvement in the performance of facial recognition algorithms through the use of ‘Deep Convolutional Neural Networks’ (DCNNs; e.g. [6–8] see [9]. One study tested algorithms made in 2015, 2016 and 2017 and showed a monotonic increase in performance from the oldest (68% accurate) to the newest (96% accurate [10]). The National Institute of Standards and Technology (NIST) in the USA runs a regular Face Recognition Vendor Test (FRVT) which is a standard test of facial recognition algorithms. The FRVT has consistently reported improvements in algorithm 1:N face identification and now conducts continual testing of algorithms and produces a publicly available ranking of their performance (e.g. [11]). The algorithm currently topping this leaderboard has a false negative rate of around or under 1% in 5 of the 8 tests (6.9%, 9.9% and 16.7% in the other three tests respectively), with a false negative rate being the percent of searches with a match in the system failing to return that matched image [12]. Overall, false negative rates of the 274 algorithms that were submitted to this most recent evaluation ranged from 0.15% to 99.99%.

In the UK, the Data Protection Act 2018 states that any identification ‘decision’ made by an algorithm must be checked by a human. Increasingly, hybrid human-AFR systems are used in 1:N identification settings where typically the human is used to verify the top matches returned by the algorithm (see [5] section 1.4 for a detailed overview). Combining algorithm and human judgements may yield the highest accuracies for the most challenging conditions including identification across changes in pose and lighting as well as identification from blurry images and videos [10]. Depending on their design, systems that integrate humans and algorithms can both enhance and reduce accuracy.

### Algorithm bias

Demographic biases in AFR have been a cause for concern in recent reports because they contravene the fundamental human right that citizens should be treated equally [13–15]. One may expect algorithms to be free from the biases that humans often show in face recognition. However, it is now known that algorthims also show bias, which can be built into algorithms as a function of the system design and programming, data, or images they are trained on. For example, face recognition algorithms show the Own Race Bias [16–18], whereby humans are typically better at remembering and comparing faces from their own demographic group opposed to another race (see [19] for review).

Another example concerns gender classification. Algorithms trained on datasets which contain mainly lighter-skinned people have been shown to produce gender classification errors of up to 34.7% in darker-skinned females compared to only 0.8% in lighter-skinned males [17]. A preprint, however, tested five commercial facial recognition algorithms and showed that most features used by these algorithms to make identity judgements were unrelated to gender and race [20]. Another study tested four algorithms (one previous generation, and three based on DCNNs) and found that race bias increased with item difficulty, and that equal levels of false acceptance rates for each ethnicity could only be achieved by changing the “decision threshold” for each race [21]. Therefore it was possible in this case to eliminate bias, but only by making acceptance decisions less strict for different races.

In 2019, NIST published the FRVT: Demographic Effects which describes and quantifies demographic differentials for modern commercially avaliable facial recognition algorithms [18]. This test of over 100 facial recognition algorithms showed large discrepancies between the performance of different algorithms. For example, while some algorithms did not show a race bias, other algorithms falsely identified non-White faces between 10 and 100 times more often than White faces. These results highlight the need for agencies using AFR to know how well their algorithm performs with different faces, and whether the threshold for identification should be kept constant across all faces.

The type of bias described above involves differential accuracy for one demographic group relative to another. However, other types of bias introduced by AFR are also important to consider. For example, when people are presented with prior face identification decisions that have been made by algorithms or humans, this can bias their face matching decisions [22–24]. This suggests that human-algorithm hybrid systems which require the human to verify a decision made by an algorithm may be open to the human biasing their decision in the direction of the algorithm decision. This is likely to amplify any existing biases based on differential accuracy for demographic groups.

### Use and governance

AFR has been integrated with CCTV and used by some police forces in the UK, the USA and Australia for a number of years, although to different extents in the different countries [25,26]. There is a lack of reliable information around the first date of AFR use, and the pervasiveness of AFR use in the UK, USA, Australia and China, and so instead of providing such information here, we focus on broad definitions and legal use cases. AFR is typically used by the police to match the digital representations captured by the technology with images present in a database [4]. In theory, this database could contain images of every citizen, or only images of individuals on a ‘watchlist’ [27]. Watchlists are created by authorities and contain information about a person of interest, typically fugitives and those deemed to require close surveillance [27]. Trials of live AFR deployed on city streets by police in the UK have reported high numbers of incorrect matches (i.e., false positives; [27,28]).

The use of images as evidence in legal proceedings tends to be more obviously regulated–by various rules of evidence and procedure (e.g. PACE (1978) in England and Wales), but these are hardly consistent or principled. Before AFR became quite accurate (in specific conditions) and capable of outperforming humans, most courts allowed jurors to examine images–often of a crime, such as an armed robbery–and to compare the images with images of the defendant as well as the appearance of the defendant in court. In many cases this lay comparison was supported by the opinions of police officers–often those investigating the crime–and/or a range of putative experts, sometimes described as facial mappers. These ‘mappers’ originated from a wide range of domains–specialist police, anatomy, IT, photography, military intelligence, art, anthropology–but were unified by their pervasive inattention to validation, accuracy and cognitive bias [29,30].

English courts admitted the opinions of investigating police and those recognised as experts, and allowed the examiners to make claims about similarities as well as categorical identifications [31]. Australian courts, in contrast, prevented police officers from making identifications and recently appear to have deemed the opinions of mappers (at least where offenders are well disguised) inadmissible [32,33]. The disparate jurisdictions of the USA have been influenced by Daubert v Merrell Dow Pharmaceuticals, Inc. [34] and the need for (validity and) reliability, in conjunction with emerging concerns about the forensic sciences [35,36]. Though, express concern with reliability as an admissibility pre-condition has not prevented reliance on a range of mappers. The admissibility of an identification by AFR has yet to be considered by a court in the jurisdictions considered in this survey. With the improved accuracy of the latest generation of algorithms, the admission of the output of an algorithm (as ‘machine testimony’, see [37]) or the combination of human/AFR systems can only be a matter of time [38].

The governance of AFR differs greatly across countries. The UK has a surveillance camera commissioner, a government-appointed position, and a surveillance camera code [39]. There is no equivalent in the USA. In Australia, an Identity-matching Services Bill (2019) is being considered by parliament which would allow the use of AFR to assist with identity verification by government and industry for transactions with citizens and customers, and also to identify suspects in criminal investigations. As in other social democracies, human rights and civil liberties organisations have expressed concern about the expanding use of AFR, especially dangers identified in the USA and UK with race and bias [40].

The increased use of AFR, combined with the lack of clear legislation around its use, and the potential for bias and consequential errors, has led to debates around the ethics of gathering face images for training algorithms, and the use of AFR by state and private users [3,41]. There have also been high profile calls for the outright banning of AFR by public interest groups such as banfacialrecognition.com and Big Brother Watch. Recently an independent research institute called for a moratorium on AFR following its survey of public opinion in the UK [13], and The Electronic Privacy Information Center (EPIC) issued an open letter, signed predominantly by organisations in the USA, opposing the use of AFR by private companies as well as governments [42]. As well as calls for bans, there have been several challenges to the use of AFR. In the UK, South Wales Police’s use of AFR was ruled as unlawful as a breach of article 8 of the European convention on human rights [43]. In the USA, a number of cities have placed bans of varying severity on the use of AFR, and legislation currently under consideration in the USA (at the time of writing) would prohibit the use of AFR by the Federal Government [44]. In addition, three recent congressional hearings in the USA examined AFR’s impact on civil rights and liberties, and transparency in both government and commercial use, and accuracy [45–47].

The London Policing Ethics Panel in 2019 made three recommendations around the use of live AFR: that there should be enhanced ethical governance of policing technology field research trials; that public views on live AFR should be reviewed after the deployment of live AFR; and that there is a need to simplify and strengthen regulation of new identification technologies [15]. Similarly, the UK Information Commissioner gave an opinion on the use of live facial recognition technology by law enforcement in public places which concluded that the use of live AFR should meet the threshold of strict necessity. For example, to locate a known terrorist but not to be used indiscriminately in order to identify suspects of minor crimes, that the government should introduce a code of practice, and that public debate around the use of AFR should be encouraged [14]. The mention of engagement with the public and surveying public opinion which is common to both these institutions’ recommendations highlights the need to understand what the public know and think about the use of AFR.

### Public opinion

Given the wide use of facial recognition technology in society and recent mass media attention, it is surprising that there are relatively few publicly available surveys of public opinion, and no comparisons of public opinion across different jurisdictions.

A recent survey of public opinion in the UK asked participants about the use of AFR by police, government, and private sector, in airports, on public transports, in schools, in supermarkets and by human resources departments in workplaces. This survey showed that 46% of people thought the public should be given the ability to consent to or opt out of the use of AFR, 55% agreed the government should limit police use of facial recognition technology, and that support for the use of AFR by police (70%) was higher than in airports (50%) or in supermarkets (7%), schools (6%) and at work (4%) [13]. This survey reveals differences in attitudes to the use of AFR by different users and for different use cases. Another survey of Londoners found that support for police use of live AFR was also dependent on the use case, with 81–83% support for serious crimes depending on the nature of the threat, compared to 55% support for minor crimes, and below 50% for nuisance behaviour [15].

In Australia, half or respondents to a public opinion survey believed that the use of AFR in public spaces constitued an intrusion of privacy, but consistent with the UK, there was public support for particular uses of the technology and especially for policing [48]. A recent survey by Beijing News Think Tank found that over 80% of Chinese people surveyed opposed the use of AFR in commercial zones in Beijing, and 96% were worried about security around personal information and data [49].

### The current study

In the present studies, we sought to explore, understand and compare the attitudes towards AFR of members of the public in Australia, the UK, the USA and China, with emphasis on criminal justice applications of AFR. We focussed particularly on differences in public opinion depending on the people or group that was deploying and using the technology (users) and the specific purpose for which it is being used (use cases). We began with focus groups aimed at finding themes, or common areas of discussion (Study 1) which we then further explored in a large-scale international survey (Study 2).

The conversations in the focus groups (Study 1) fell into three overarching themes: society, technology, and purpose. Participants in all countries (Australia, China, USA) generally spoke about the same things, with notable differences being that people in China spoke more about current uses of AFR, and people in China and Australia thought of AFR as more accurate than in the UK. These differences likely reflect different uses of AFR and different reporting in the media across the different countries. Again response to the questionnaire (Study 2) were similar across countries (Australia, UK, USA) with some notable differences whereby people in the USA were more accepting of AFR being used to track citizens, and more accepting of use by private companies. Key issues surrounding privacy, trust, and a need for legislative boundaries around the use of AFR came up across both studies, as did differing levels of acceptance depending on who AFR was being used by and for what purpose.

## Study 1—Focus groups

### Methods

#### Ethics statement

Both studies presented here were given ethical approval from the University of Lincoln Research Ethics Committee (Project ID 449) in accordance with local and international regulations. All participants gave written or electronic informed consent.

#### Participants

Focus groups were conducted in the UK, Australia and China. Two focus groups were conducted in each country, with each group comprising between 7 and 11 participants. In total, 58 people took part in the focus groups. The demographic data collected was inconsistent across countries due to a miscommunication within the research team. In Australia, 18 participants (no age data; 10 male, 8 female) took part. All indicated having heard of AFR, and 55% reported feeling they were knowledgeable about AFR prior to the focus groups. In China, 20 participants took part (no age or gender data), 90% had heard of AFR and 20% felt knowledgeable about AFR prior to the focus groups. In the UK, 20 participants took part (mean age 38 years; age range 20–70 years; 3 male, 16 female, 1 no gender response), 90% had heard of AFR and 10% felt knowledgeable about AFR prior to the focus groups. All focus group members were given £30 (or the local equivalent) to compensate them for their time.

#### Procedure

The focus groups took place between 23^rd^ May 2019 and 12^th^ July 2019. Focus groups were recorded and transcribed. The schedule of questions was translated into Chinese for the two focus groups which were conducted in China. Those focus groups were conducted in Chinese, and the transcripts were translated back into English by the focus group moderators (who are fluent in both Chinese and English) for analysis. Focus group moderators gave prompt information and questions to begin the discussions, and while some of the themes identified, for example ‘accuracy’ and ‘who uses it’, were closely linked to the questions, other themes such as trust and privacy were evident in all focus groups without being explicitly prompted. There were nine prompt questions covering background knowledge of AFR, how participants would feel about it being used in different situations, and accuracy. The full schedule of focus group questions is in S1 File. All sessions lasted no longer than one and a half hours.

#### Analysis

Two researchers independently coded the transcripts by hand and conducted a collaborative thematic analysis to explore the data and generate key themes. The team followed the six phases of analysis outlined in [50], initially familiarising themselves with the data and generating codes. Potential themes were then identified and reviewed to establish three overarching themes. These overarching themes were identified and named to encompass each of their respective themes and subthemes. All of the themes and subthemes were represented in the analysis, and both researchers were in complete agreement in terms of the themes identified.

### Results

**Fig 1:**
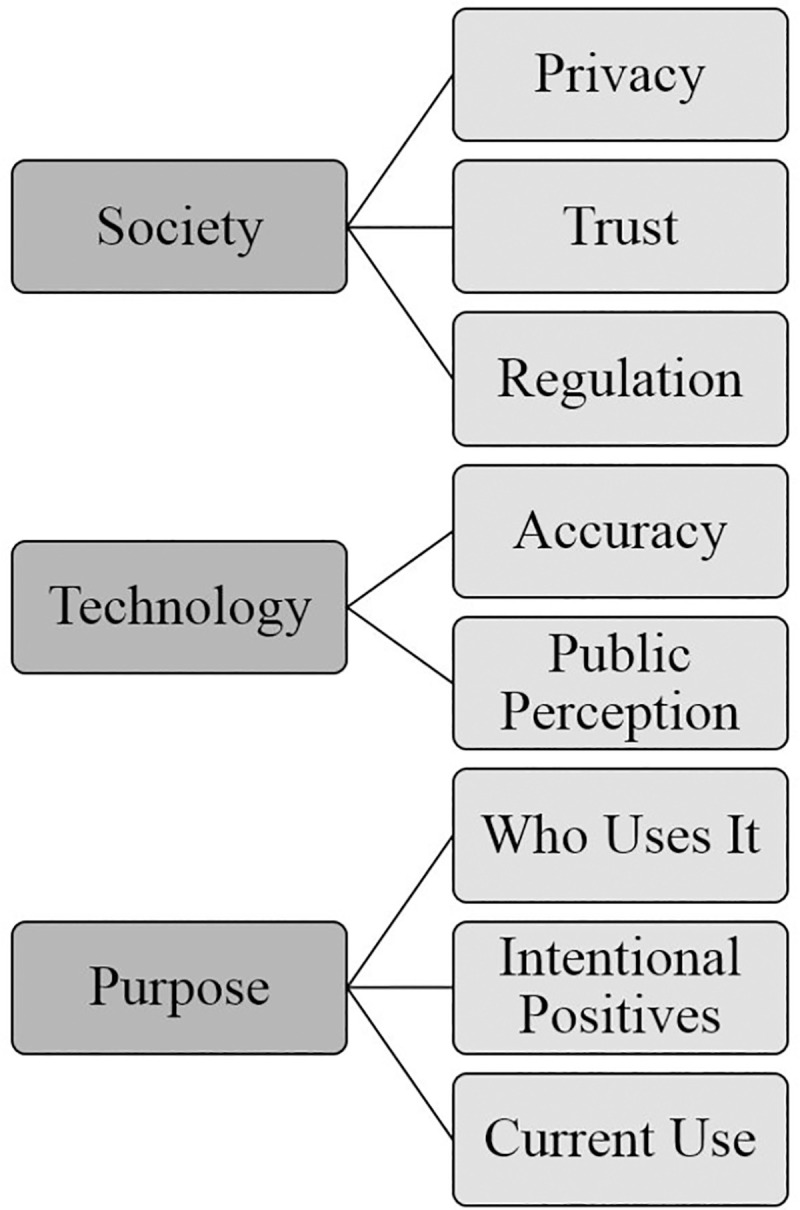
Focus group overarching themes. Graphical representation of overarching themes and themes identified from focus groups conducted in the UK, Australia and China.

**Fig 2:**
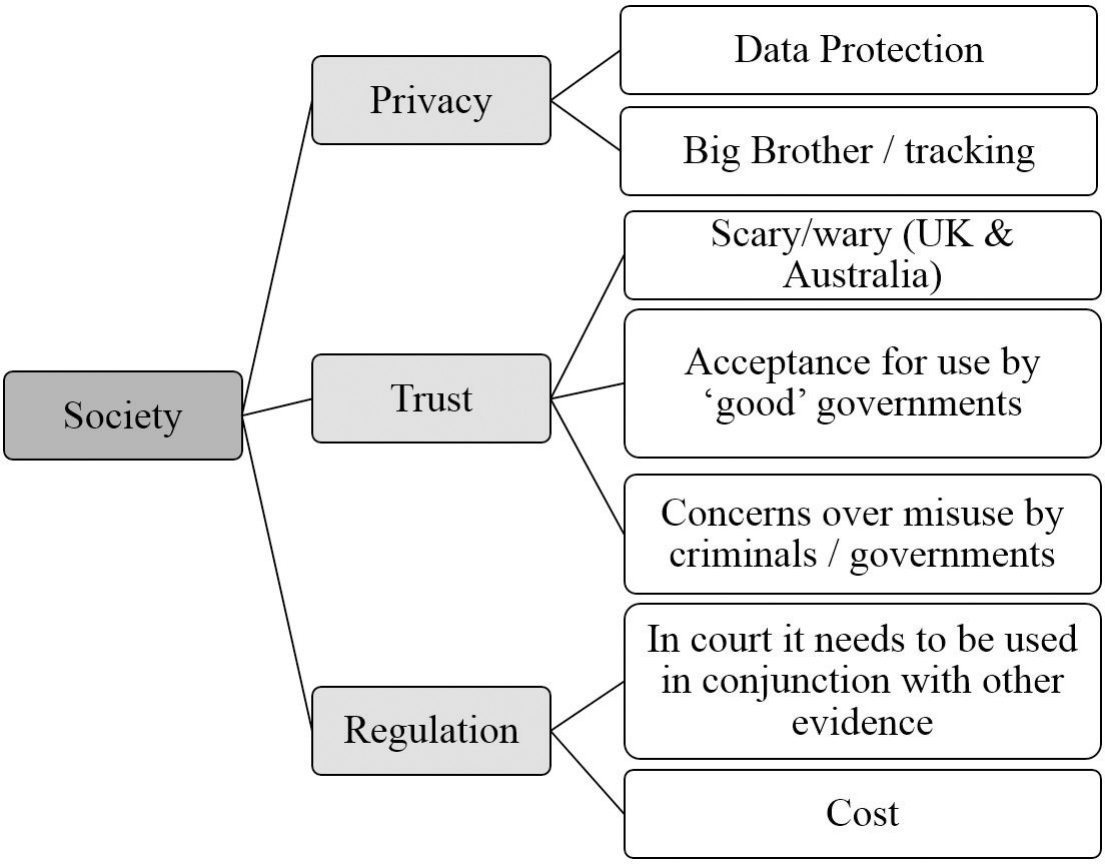
Society overarching theme. Graphical representation of Society overarching theme and its component themes and subthemes identified from focus groups conducted in the UK, Australia and China.

**Fig 3:**
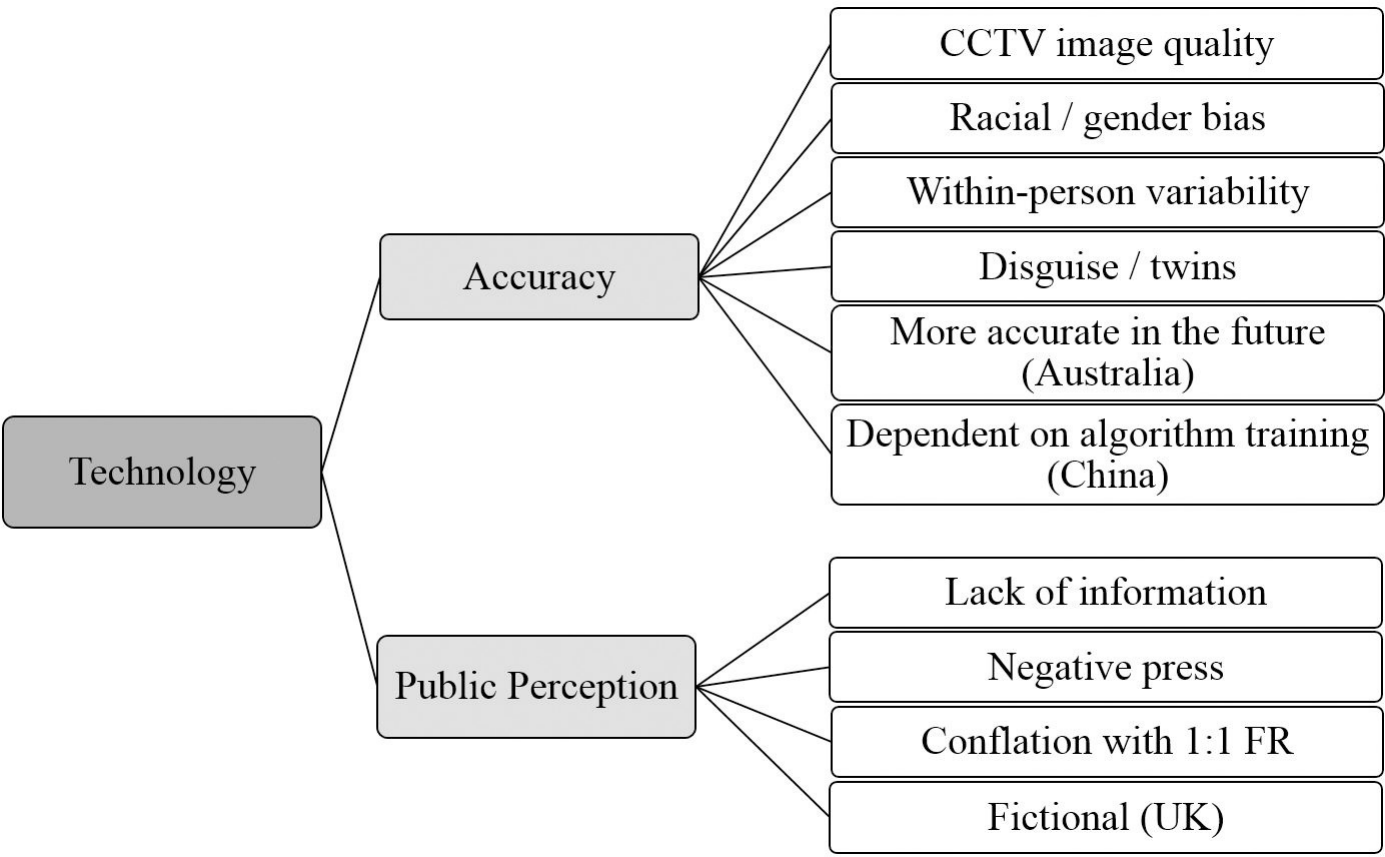
Technology overarching theme. Graphical representation of Technology overarching theme and its component themes and subthemes identified from focus groups conducted in the UK, Australia and China.

**Fig 4:**
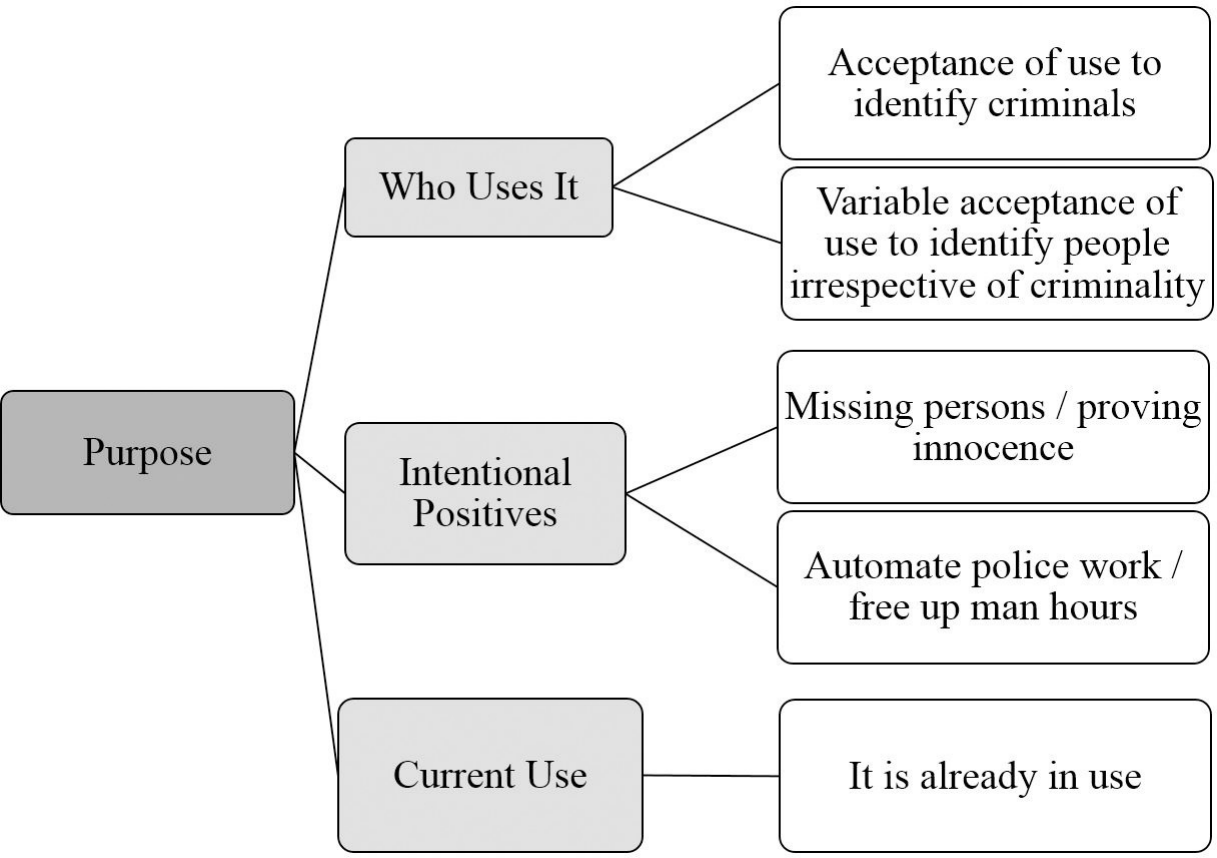
Purpose overarching theme. Graphical representation of Purpose overarching theme and its component themes and subthemes identified from focus groups conducted in the UK, Australia and China.

The overarching themes, themes and subthemes identified during the thematic analysis are shown in Figs 1–4.

All themes that were identified were common across all countries, but some sub-themes were specific to pairs of countries. Here, we describe all subthemes in turn.

#### Society—Overarching theme

Fig 2 shows the Society overarching theme with each of its component themes and subthemes.

*Privacy theme*: *Data protection subtheme*. Participants were concerned about the storage and sharing of data, including images of their face.

*Privacy theme*: *Big Brother/tracking subtheme*. Participants were concerned about the use of AFR to track individuals, and felt that this could normalise surveillance.

*Trust theme*: *Scary/wary subtheme*. This subtheme was specific only to the UK and Australia and did not come up in the Chinese focus groups. People were wary of AFR and expressed being scared by it. Participants in the first UK focus group made comments such as “It is just terrifying” and “How it is being used in China I think is absolutely petrifying”, and in one Australian focus group made comments such as “It’s innocent until it’s not”.

*Trust theme*: *Acceptance for use by ‘good’ governments subtheme*. Focus group participants in all three countries seemed confident that their own government was ‘good’ and would use the technology responsibly, but were concerned that it should not be used in other countries whose governments they trusted less. One participant in Australia said “It’s like the example that comes to mind is like what’s happening in China there’s obviously a bit of an authoritarian police state emerging or it’s pretty much there. I think in Australia I’d be more comfortable with it because the justice system is (like) just”, and a participant in China said “For example, I think our country may be okay, but for the United States, it may be easier to deepen this kind of judicial bias for those who are [non-White] or marginalized”.

*Trust theme*: *Concerns over misuse by criminals/governments subtheme*. Participants in all three countries expressed concern that in the wrong hands, the technology could be misused.

*Regulation theme*: *In court it needs to be used in conjunction with other evidence subtheme*. Participants in all three countries felt comfortable with AFR being used as evidence in courts, but all expressed that this should only be used in conjunction with other evidence.

*Regulation theme*: *Cost subtheme*. Cost was mentioned in all countries. Participants showed an understanding that the technology would require financial investment from governments/police forces/local authorities to set up and use. There was also concern that without large monetary investment, the systems may not be as accurate as they could be.

#### Technology—Overarching theme

Fig 3 shows the Technology overarching theme with each of its component themes and subthemes.

*Accuracy theme*: *CCTV image quality subtheme*. In all three countries, participants were concerned that AFR would not work well if used with poor quality CCTV images. Particularly in the UK there was a concern that all CCTV images are poor quality. This is not the case, and in fact all focus groups were conducted in Lincoln which had recently undergone a CCTV upgrade with very high quality cameras in use since 2018 [51].

*Accuracy theme*: *Racial/gender bias subtheme*. We did ask a specific question about perceptions of any racial bias in AFR (see S1 File), but this came up in each focus group prior to the question being raised with the group. Participants had seen news reports about potential inaccuracies of AFR for non-White people. This was mentioned in the UK and Australian focus groups, but not the Chinese focus groups.

*Accuracy theme*: *Within-person variability subtheme*. In all countries, participants were concerned that AFR may not be able to cope with changes in appearance. Makeup and changes of hairstyle were mentioned in all focus groups. This leads on to the next subtheme.

*Accuracy theme*: *Disguise/twins subtheme*. Participants in all countries spoke about the use of disguise to evade AFR, and the possibility that AFR would not be able to discriminate between similar looking people, for example twins.

*Accuracy theme*: *More accurate in the future subtheme*. This was discussed in both Australian focus groups, but not in the UK or China. This may be due to the fact that people living in Australia may be more concerned about AFR because the enactment of the Identity-matching Services Bill (2019) which would see AFR used on a national scale has led to more media coverage of, and perhaps more public interest in AFR. Participants in Australia, when asked about accuracy, felt that although AFR may not be perfectly accurate at the moment, it would continue to develop and would become more accurate in the future.

*Accuracy theme*: *Dependent on algorithm training subtheme*. This was only discussed in our Chinese focus groups, perhaps due to the more widespread use of AFR in China, leading to a greater public knowledge of how the systems work. When asked about accuracy, participants in China were aware that the success of a system depends a great deal on the images with which it is trained.

*Public Perception theme*: *Lack of information subtheme*. Participants in all of our focus groups agreed that there is a lack of information in their country about how AFR systems are built, how they are used, and how the data are stored and shared.

*Public Perception theme*: *Negative press subtheme*. Focus group participants in the UK and Australia, but not China, stated that they had seen negative press surrounding their country’s use of AFR.

*Public Perception theme*: *Conflation with 1*:*1 facial recognition subtheme*. In all of our focus groups, there was at least one discussion in which participants conflated the 1:N identification use case with the 1:1 verification. This is important to note because participants had been given a definition of 1:N identification as opposed to 1:1 identity verification, and so this confusion between the two may reflect a lack of distinction between these two processes in the general public, and in how this technology is described.

*Public Perception theme*: *Fictional subtheme*. Perhaps the most surprising subtheme was mentioned only by participants in the UK and Australia, but was mentioned frequently. Some people expressed that they did not really believe AFR was real, and they had only come across it in films or on TV. One UK participant said “I have only really come across it in television programmes…so it has always been something that I kind of almost didn’t really necessarily think was real”, and one participant in Australia asked “Is it already out there?”.

#### Purpose—Overarching theme

Fig 4 shows the Purpose overarching theme with each of its component themes and subthemes.

*Who uses it theme*: *Acceptance of use to identify criminals subtheme*. Participants in all focus groups were mostly accepting of the idea that AFR could be used to identify people who had committed crimes.

*Who uses it theme*: *Variable acceptance of use to identify people irrespective of criminality subtheme*. In contrast with the previous subtheme, participants in all focus groups were less accepting of the idea that AFR could be used to identify anyone, irrespective of whether or not they had committed a crime. There was, however some disagreement on this. In the UK one participant said “You could be innocently out with your family or on your own or anything and then this facial recognition could pick you up”, but another said “I just think if you’re not doing anything wrong then why would you have a problem with it”. These sorts of disagreements came up in all focus groups, and are a regular feature of debates over the expansion of surveillance and crime control in recent decades.

*Intentional positives theme*: *Missing persons/proving innocence subtheme*. The intended positives of AFR came up in all focus groups. Most frequently people mentioned that it could be used to locate missing persons, or that if you were accused of a crime but were not actually present, it could show that you were somewhere else at the time. These uses, particularly the second, may be of limited value given the increased scope of constant surveillance of all people in public places which would be required.

*Intentional positives theme*: *Automate police work/free up man hours subtheme*. Participants in all focus groups also felt that a positive aspect of AFR is that it could automate some aspects of police work, and that it could free up man hours spent searching for people in CCTV footage.

*Current use*: *It is already in use*. Participants in Australia noted that AFR is already in limited use in their country, and that acceptance of use in one scenario could lead to more widespread use, saying things such as “…it’s a slippery slope…”. In China participants spoke of the current widespread use of AFR for payments and for access to University accommodation. Participants in the UK did not speak of current AFR use in their country.

### Discussion

In general, the themes covered by members of all six of our focus groups across all three countries were similar. People in all three countries were concerned about privacy, trusting the users of AFR, and thought the use of AFR should be regulated. Notable differences were that participants in China spoke more about current AFR use in their country, and this was not spoken about in the UK. This may reflect differences in use of AFR in these countries (with China reportedly using AFR frequently in many public spaces, and only a small number of UK police forces trialling AFR use), and so shows that our participants were sensitive to AFR use, or perhaps media commentary, in their own country. Other differences were that participants in Australia and China tended to think of AFR systems as being accurate, where participants in the UK thought of it as less accurate. This may reflect differences in media reporting, or stories picked up by the press in these countries. Using the themes that had been identified in Study 1, we created specific questions for our large-scale questionnaire. We aimed to survey a large number of people in different countries to gain insight into public attitudes towards the use of AFR in different criminal justice systems.

## Study 2—Questionnaire

The questionnaire questions were predominantly derived from the themes that were identified in the focus groups. In addition, we replicated some questions from the Ada Lovelace Institute report [13].

Prior to finalising the questionnaire, we sought feedback from a variety of sources. We initially sought feedback from members of a multidisciplinary group who attended a meeting of academics, police, forensic services and related industries–the Unfamiliar Face Identification Group (UFIG). We surveyed members of UFIG2020 (those attending the 2020 iteration of the group meeting) via an online survey link prior to the meeting. We received 26 responses, with 15 respondents identifying themselves as academics/researchers, 3 as members of the police/forensic services, and 8 as ‘other’ including public servants and biometrics suppliers, and federal government. Respondents were given a list of the intended topics to be covered in the questionnaire and asked to indicate to which (if any) they would be interested or uninterested in knowing public responses. We included space for respondents to make suggestions, but none were made. Our intended topics were looked upon favourably by this group (*M* = 74% interested in each topic; *M* = 4% not interested in each topic). The questions were then sent in full to the Ada Lovelace Institute who acted as an independent body to verify that the specific wording of the questions was unbiased and not leading.

We collected data from Australia, the UK, and the USA. We added the USA here so as to compare three Western, English-speaking countries which all use AFR to different extents. Initially we had intended to collect data from China, but found we could not access participants in China through either of the data collection websites we used (MTurk and Prolific.co). We collected data from participants in India via MTurk, but do not report those data here due to concerns about data quality and internal consistency of responses on reverse-coded questions (specifically questions 13–15).

### Methods

#### Participants

Australia: We collected data from people currently living in Australia via a combination of MTurk (all Mturk participants in all countries were given USD$1.14 or local currency equivalent as compensation for their time), Prolific.co (all Prolific.co participants in all countries were given GBP£1.00 or local currency equivalent as compensation for their time), and a mailing list of interested participants maintained by the University of New South Wales (mailing list participants volunteered without monetary compensation for their time). The final sample consisted of 1001 participants (557 female, 439 male, 5 other or not disclosed; mean age 40 years, age range 16–82 years; 532 White, 174 non-White, 295 not disclosed). Note participants were coded as White or non-White according to their response to a free text entry question asking “What is your ethnicity?” Participants who entered a country of residence, or another response from which their ethnicity could not be ascertained (e.g. “Australian”) were not included in the ethnicity breakdown of results (see data in Supporting Information S4–S6 Files). This was applied to data from all three countries.

UK: We collected data from people currently living in the UK via a combination of MTurk and Prolific.co. The final sample consisted of 1107 participants (620 female, 483 male, 4 other or not disclosed; mean age 34 years, age range 16–82 years; 793 White, 143 non-White, 171 not disclosed).

USA: We collected data from people currently living in the USA via MTurk. The final sample consisted of 1016 participants (432 female, 579 male, 5 other or not disclosed; mean age 38 years, age range 19–74 years; 700 White, 252 non-White, 64 not disclosed).

### Procedure

The questionnaire took around ten minutes to complete and was split into five sections: 1) background knowledge; 2) use; 3) trust; 4) use in court; 5) accuracy. Each section contained multiple questions aimed at addressing different aspects of people’s attitudes towards AFR. Question text is given in S2 File, and full questions and data are available in the data in S4–S6 Files. This includes details for each question as to whether multiple responses were allowed. Participants were not given the option to skip any questions, but ‘other’ or ‘I don’t know’ answer options were included. All questions which required participants to rate agreement with a statement or rate trust or comfort etc. used a 6-point scale. Previous research has suggested that including a midpoint in a scale can lead to over-selection of that middle option [52], particularly in non-Western populations [53]. This was particularly important as we had initially intended to have responses from India and China as well as Australia, the UK and the USA. Therefore we did not use a scale midpoint, allowing us to split data into those who did not agree/trust etc (those responding 1, 2 or 3) and those who did agree/trust etc (those responding 4, 5 or 6) without any loss of data. All such questions were presented as a six-point scale with anchoring statements only on the first and last points, for example “To what extent do you agree with facial recognition technology being used by the police in your country in their day to day policing? Please answer using the scale 1 do not agree at all to 6 strongly agree”. We also included data quality/screening checks (see S3 File for full details). All data were collected simultaneously across all countries between 28^th^ December 2019 and 29^th^ January 2020.

### Results and discussion

The full data can be accessed in S4–S6 Files. There the data are broken down by age groups, sex, ethnicity, region (urban/rural), and educational level. Here we present only the total responses across all participants in order for us to compare across the three countries (Australia, UK, USA). Data for key comparisons was analysed using z tests, which while appropriate for independent samples (e.g. differences between responses from different countries), gives more conservative results when used with related samples (e.g. differences between all participants’ responses to different use cases).

Although there was broad agreement across countries, responses from participants in the UK and Australia were more similar to each other, with responses from the USA differing in some interesting ways. The questionnaire data are broken down into five sections: 1) background knowledge; 2) use; 3) trust; 4) use in court; 5) accuracy. We will address the main points from each of these sections in turn.

#### Section 1: Background knowledge

Most importantly from this section, a mean of 90.93% of participants stated that they were aware of facial recognition as a method of identity verification (Q1 Australia: *M* = 95.41%, UK: *M* = 93.13%, USA: *M* = 84.25%); 28.39% stated that they currently use facial recognition as a method of identity verification (Q2 Australia: *M* = 30.37%, UK: *M* = 32.25%, USA: *M* = 22.54%); and 42.59% stated that in an ideal world, they would like to rely on facial recognition as a method for identity verification (Q3 Australia: *M* = 47.55%, UK: *M* = 42.82%, USA: *M* = 37.40%). In all three countries, fingerprints (*M* = 60.52%) and passwords (*M* = 54.73%) were the most popular forms of identity verification in an ideal world.

**Fig 5:**
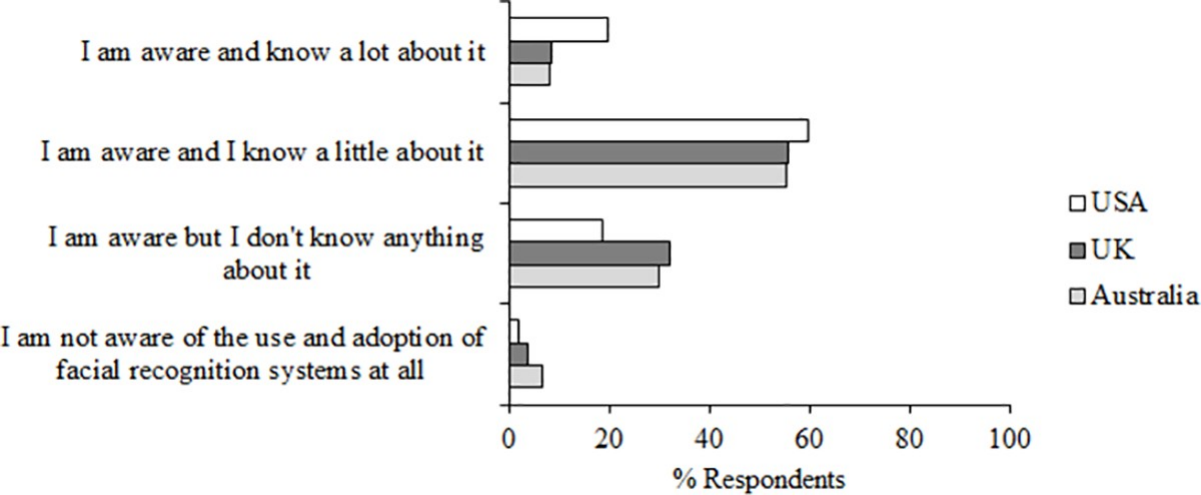
Question 4 data. Responses to question 4 “How aware are you of the use and adoption of facial recognition systems in your country?”.

Prior to indicating their knowledge of AFR, participants were presented with the following description, adapted from the Ada Lovelace Institute’s questionnaire [13]: “Facial recognition technology is a biometric system which aims to identify or observe individuals by detecting features associated with a human face. A digital representation of the face is created which can then be compared against a database of stored images. This digital representation may be used to infer characteristics of individuals, and can be matched with similar images to verify a person’s identity or uniquely identify individuals.” Importantly, participants did not indicate that they considered themselves expert in their knowledge or awareness of the adoption of facial recognition systems in their country (Q4) with a mean of 31.01% participants responding that they either are aware but don’t know anything about it, or are not aware of the use and adoption of facial recognition systems at all. Fig 5 shows the percent of respondents from each country who chose each option in response to this question.

#### Section 2: Use

**Fig 6:**
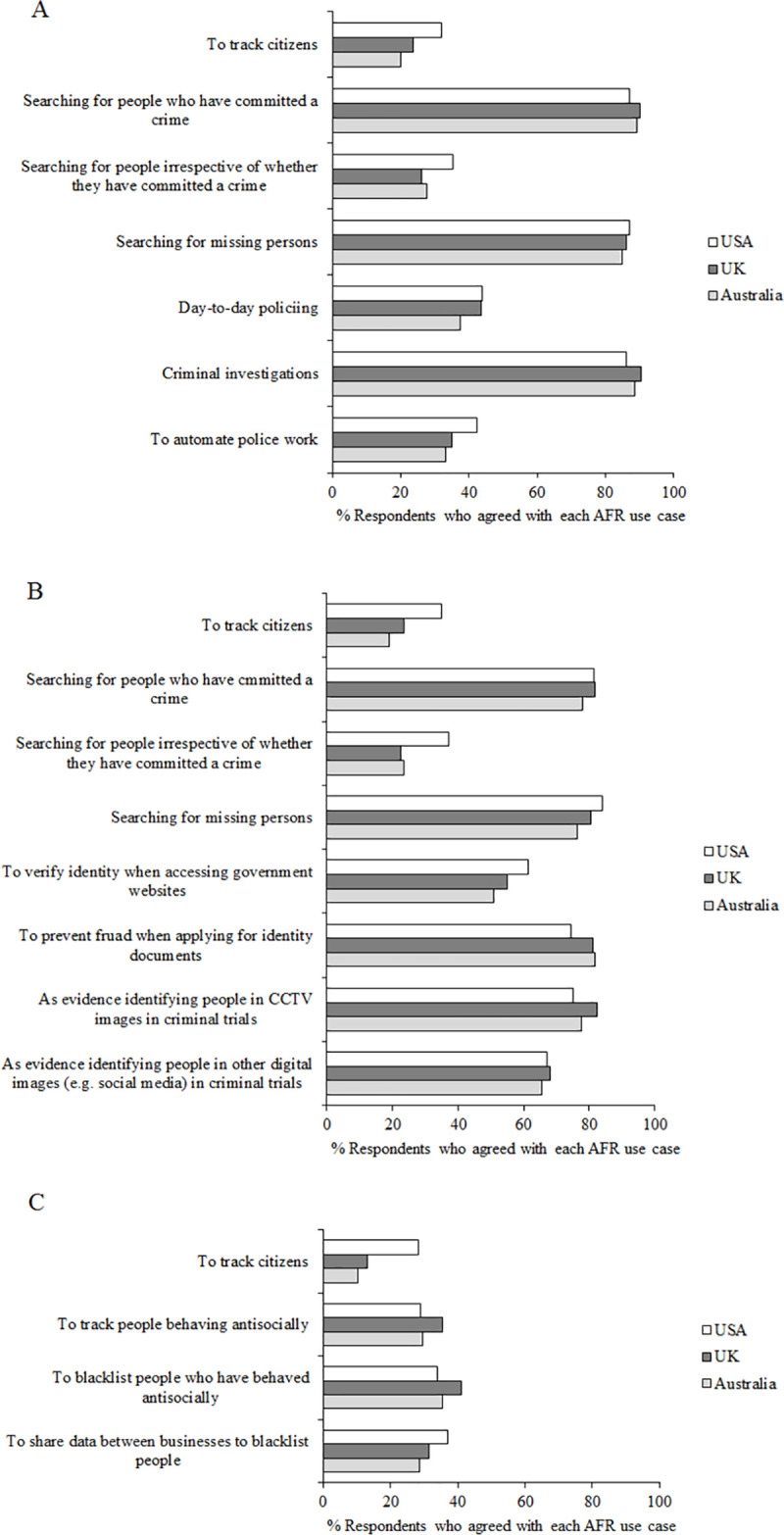
Questions 5–7 data. Percent of participants who agreed (responding 4, 5 or 6 on the scale of agreement) to questions 5–7 “To what extent do you agree with facial recognition technology being used by the police/the government/private companies in your country?” for each use case. **A**, police. **B**, government. **C**, private companies.

In this section we asked questions about different actual and potential uses of AFR a) by the police, b) the government, and c) private companies. For each question, participants were asked to think about the police, government or private companies in their own country. Fig 6 shows responses to questions 5–7.

Participants were generally more accepting of police and government use of AFR than use by private companies. One use case—to track citizens—was presented for all three organisations (police, government, private companies), and across all countries the agreement with this was low (*M =* 22.77%), but higher for police (*M* = 25.31%) and governments (*M* = 25.80%) than private companies (*M* = 17.21%; private companies compared to police z = 7.86, *p* < .001, df = 6,246; private companies compared to governments z = 8.31, *p* < .001, df = 6,246). Comparing police and government use for the same use cases, agreement was similarly low for police and government using AFR to track citizens, and to search for people irrespective of whether or not they have committed a crime (police: *M* = 29.61%, government: *M* = 27.60%), but there was greater agreement for police using AFR both to search for people who have committed a crime (police: *M* = 88.86%, government: *M* = 80.42%) and to search for missing person (police: *M* = 86.06%, government: *M* = 80.25%).

Agreement with police use was high for searching for people who have committed a crime, searching for missing persons, and use in criminal investigations (*M* = 88.42%), and was lower for use to track citizens, searching for people irrespective of whether or not they have committed a crime, in day-to-day policing, which could be akin to trawling (*M* = 41.69%), and to automate police work (*M* = 36.88%).

Agreement with government use was low for tracking citizens, and searching for people irrespective of whether or not they have committed a crime. Just over half of participants agreed that the government should be able to use AFR to verify identity when accessing government websites (*M* = 55.72%). Agreement was higher with all other government uses of AFR: searching for people who have committed a crime, searching for missing persons, to prevent fraud when applying for identity documents (*M* = 78.99%), as evidence identifying people in CCTV images in criminal trials (*M* = 78.37%), and as evidence identifying people in other digital images (e.g. social media) in criminal trials (*M* = 66.86%).

Agreement with private companies’ use of AFR was generally low for all use cases presented: to track citizens, to track people behaving antisocially (*M* = 31.31%), to blacklist people who have behaved antisocially (*M* = 36.71%), and to share data between businesses to blacklist people (*M* = 32.21%).

Interestingly there were clear differences between participants in the USA, and those in both the UK and Australia. More participants in the USA agreed with the use of AFR to track citizens, crucially across all three users (police: USA *M* = 32.09%, Australia *M* = 20.18%, z = 6.14, *p* < .001, df = 2,015; USA, UK *M* = 23.67%, z = 4.33, *p* < .001, df = 2,121; government: USA *M* = 34.94%, Australia *M* = 18.88%, z = 8.27, *p* < .001, df = 2,015; USA, UK *M* = 23.58%, z = 5.77, *p* < .001, df = 2,121; private companies: USA *M* = 28.15%, Australia *M* = 10.39%, z = 10.39, *p* < .001, df = 2,015; USA, UK *M* = 13.10%, z = 8.66, *p* < .001, df = 2,121). More participants in the USA also agreed with the use of AFR to search for people irrespective of whether or not they have committed a crime (police: USA *M* = 35.24%, Australia *M* = 27.57%, z = 3.72, *p* < .001, df = 2,015; USA, UK *M* = 26.02%, z = 4.62, *p* < .001, df = 2,121; government: USA *M* = 37.01%, Australia *M* = 23.38%, z = 6.74, *p* < .001, df = 2,015; USA, UK *M* = 22.40%, z = 7.43, *p* < .001, df = 2,121).

#### Section 3: Trust

In this section we asked questions about how comfortable participants felt with police use of AFR, and how much they trusted the police, the government, and private companies in their country to use AFR responsibly.

**Fig 7:**
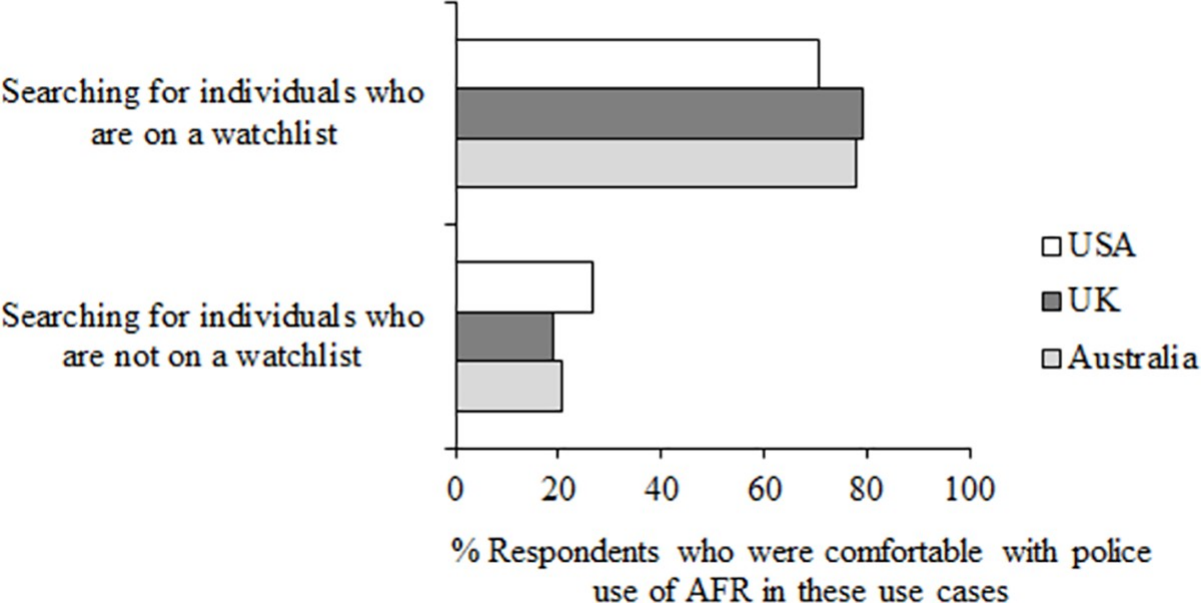
Questions 8–9 data. Percent of participants who were comfortable (responding 4, 5 or 6 on the scale of comfort) to questions 8 and 9 “How comfortable do you feel with police in your country using facial recognition technology to search for individuals who are/are not on a watchlist”.

Prior to answering these questions, participants were given the following description of AFR use: “Facial recognition technology is used by some police forces as a method of identity verification. The police may aim to match the digital representations captured by the technology with images present in a database. This database could contain images of every citizen, or only images of individuals on a ‘watchlist’. Watchlists are created by authorities and contain information about a person of interest, typically those who require close surveillance. Any images stored for this purpose must be accurate, verifiable and held lawfully by the police.” Fig 7 shows responses to questions 8 and 9.

**Fig 8:**
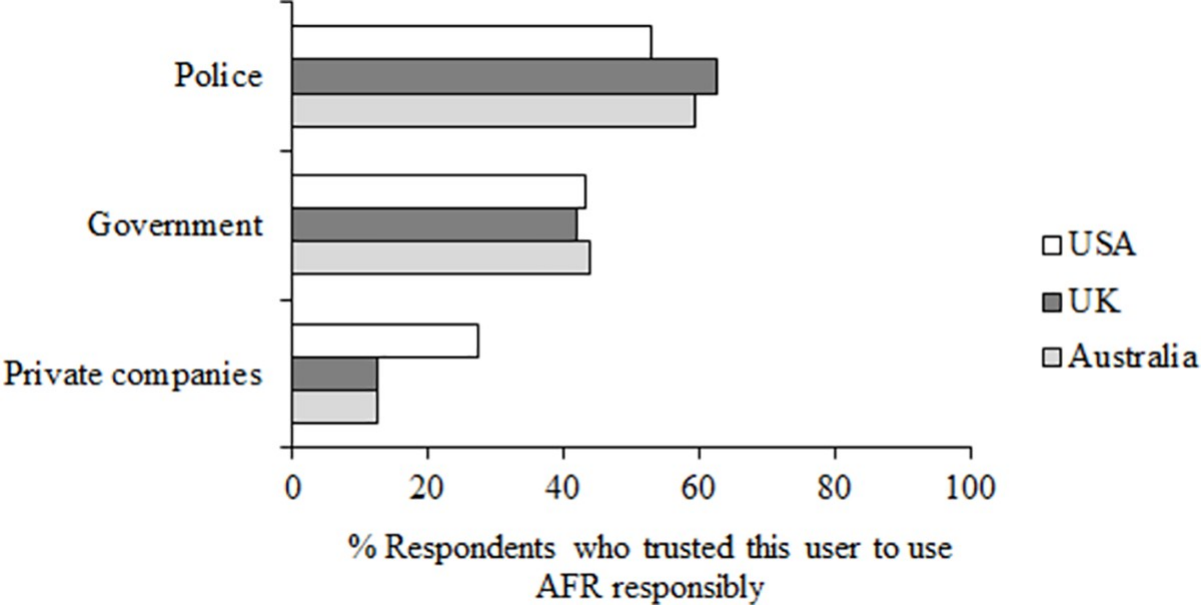
Questions 10–12 data. Percent of participants who trust (responding 4, 5 or 6 on the scale of trust) these users to use AFR responsibly, responding to the questions 10–12 “To what extent do you trust the police/the government/private companies in your country to use facial recognition technology responsibly”.

Across all three countries, most people felt comfortable with police using AFR to search for people on a watchlist (*M* = 75.81%) and a minority felt comfortable for use to search for people who are not on a watchlist (*M* = 22.13%). Again there were small differences between responses from the USA compared to both Australia and the UK. Fewer participants in the USA were comfortable with the use of AFR to search for people on a watchlist (*M* = 70.57%) than in Australia (*M* = 77.72%, z = 3.68, *p* < .001, df = 2,015) and the UK (*M* = 79.13%, z = 4.55, *p* < .001, df = 2,121). Conversely, more people in the USA were comfortable with the use of AFR to search for people who are not on a watchlist (*M* = 26.67%) than in both Australia (*M* = 20.58%, z = 3.23, *p* < .001, df = 2,015) and the UK (*M* = 19.15%, z = 4.12, *p* < .001, df = 2,121). This is consistent with data from questions 5–7 on AFR use which showed that participants in the USA were more willing for AFR to be used to track citizens irrespective of whether they had committed a crime. Fig 8 shows responses to questions 10–12.

Across all three countries, trust was highest for the police (*M* = 58.37%), then the government (*M* = 42.93%), and lowest for private companies (*M* = 17.50%; police compared to government z = 12.35, *p* < .001, df = 6,246; government compared to private companies z = 22.77, *p* < .001, df = 6,246). Although the majority of people trusted the police to use AFR responsibly (i.e. in all countries, the number of people trusting (responses 4,5,6 on the scale) compared to not trusting (responses 1,2,3 on the scale) is over 50%, so over half of the respondents), these numbers are not very high. Looking at governments, in all countries the majority of participants did not trust their government to use AFR responsibly (i.e. numbers presented in Fig 8 for trusting governments are all under 50%). In all three countries, a similar proportion of respondents indicated that they trust the government to use AFR responsibly (Australia: *M* = 43.86%; UK: *M* = 41.82%; USA: *M* = 43.11%, all z < 1, all *p* > .05), but responses differed for both the police and private companies. In the USA, trust was lower for police (*M* = 53.05%) than in Australia (*M* = 59.54%, z = 2.94, *p* = .002, df = 2,015) and the UK (*M* = 62.51%, z = 4.42, *p* < .001, df = 2,121), and in the USA trust was higher for private companies (*M* = 27.36%) than in Australia (*M* = 12.49%, z = 8.51, *p* < .001, df = 2,015) and the UK (*M* = 12.65%, z = 8.55, *p* < .001, df = 2,121).

Participants were also asked to choose from a list of reasons for their trust, or lack of trust in each user’s responsible use of AFR from a list of reasons (full details of all questions and options in the data in S2 File). Participants who responded that they did trust the user (responding 4,5,6 on the scale of trust) were only shown options as to why they did trust that user. Vice versa respondents who indicated they did not trust that user were only shown options as to why they did not trust the user. The most common reasons to trust the police, government, and private companies were “It is beneficial for the security of society”, “I trust [the user] to use the technology ethically” (more common for police and government than private companies), “The benefits to society outweigh any loss of privacy I might experience” (for government and private companies), “It is beneficial to my own personal security” (for government and private companies), and “I generally trust the police” (only for police, across all countries). The most common reasons not to trust these users to use AFR responsibly were “I am concerned about my data being misused” (most common reason in all countries for all users), “I do not trust that my data will be stored securely”, and “I do not trust [the user] to use the technology ethically” (for government and private companies).

#### Section 4: Use in court

This section of the questionnaire asked respondents about different use cases of AFR in court. At this point government organisations in the US, UK and Australia use AFR to assist with surveillance, investigations and identification. These background or investigative uses are not always clearly regulated, even though important decisions–such as denial of visas and plea deals–are often based substantially upon them. Different states and even regions in the US and Australia have adopted their own–federally uncoordinated–approaches to the admissibility of expert and ‘machine’ evidence [54].

**Fig 9:**
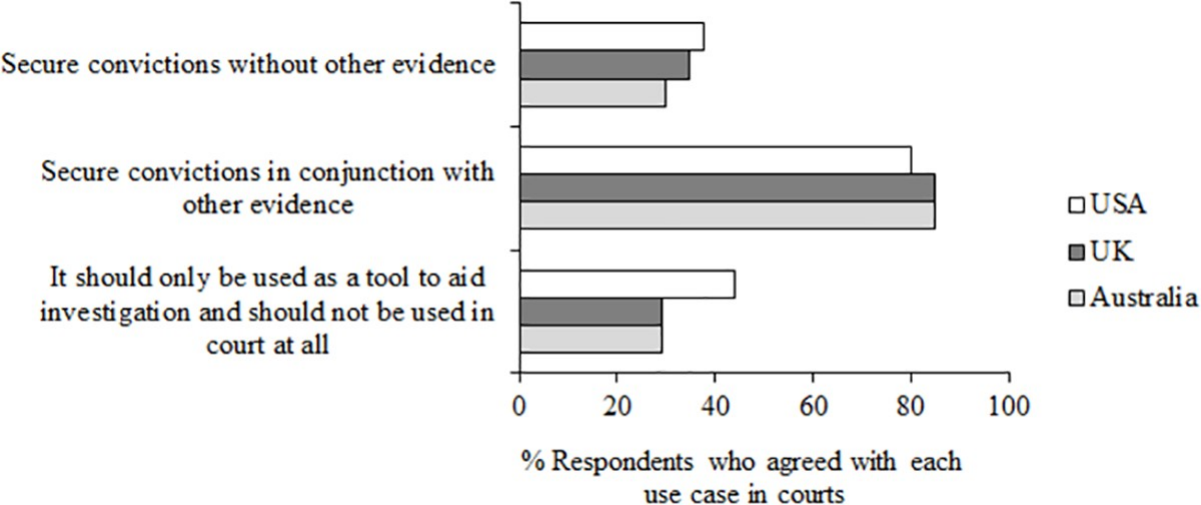
Questions 13–15 data. Percent of participants who agree (responding 4, 5 or 6 on the scale of agreement) with each use case in court (questions 13–15).

Fig 9 shows responses to questions 13–15 –“If facial recognition technology were to be used as evidence in court in your country:” Q13 “To what extent do you agree with it being used to secure convictions *without* other evidence?”, Q14 “To what extent do you agree with it being used to secure convictions *in conjunction with* other evidence?”, Q15 “To what extent do you agree that it should only be used as a tool to aid investigation and should not be used in court at all?”

Overall agreement was high for the use of AFR in court to secure convictions when it is used in conjunction with other evidence (*M* = 83.22% across three countries), and lower to secure convictions without other evidence (*M* = 34.15%) and for use only as a tool to aid investigation and not used in court at all (*M* = 34.15%; used in conjunction with other evidence compared to both used without other evidence and used only as a tool to aid investigation, both z = 45.42, both *p* < .001, both df = 6,246). Interestingly, agreement with the last and most conservative statement was higher in the USA (*M* = 34.15%) than both Australia (*M* = 29.17%, z = 2.41, *p* = .008, df = 2,015) and the UK (*M* = 29.18%, z = 2.46, *p* = .007, df = 2,121). This may reflect the USA respondents’ relatively lower trust in the use of AFR by the police (as evaluated in Question 10, see Fig 8), and so less acceptance for AFR in court.

#### Section 5: Accuracy

**Fig 10:**
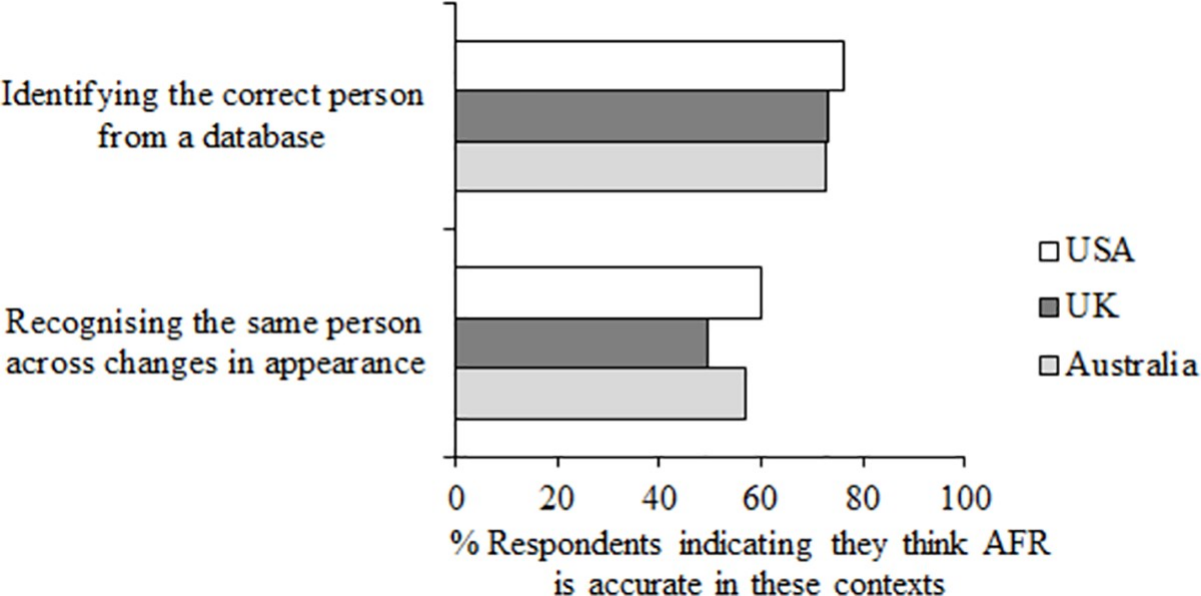
Question 16–17 data. Percent respondents responding accurate (4, 5 or 6 on the scale of accuracy) to question 16 “How accurate do you think this technology is at identifying the correct person from a database?” and 17 “How accurate do you think this technology is at recognising the same person across changes in their appearance?”.

The final section of the questionnaire asked about public perceptions of the accuracy of AFR. Prior to the first questions in this section, participants were presented with the following statement “Thinking about facial recognition technology which searches for a target person through databases of images containing multiple different people, please answer the following questions.” Fig 10 shows responses to questions 16 and 17.

The majority of respondents in all three countries thought that AFR is accurate at identifying the correct person from a database (*M* = 74.09%). Only a small majority overall, however, thought AFR is accurate at recognising the same person across changes in appearance (*M* = 55.62%). Here participants in the UK thought AFR was less accurate (*M* = 49.68%) than participants in Australia (*M* = 57.14%, z = 3.44, *p* < .001, df = 2,106) and the USA (*M* = 60.04%, z = 4.82, *p* < .001, df = 2,121).

**Fig 11:**
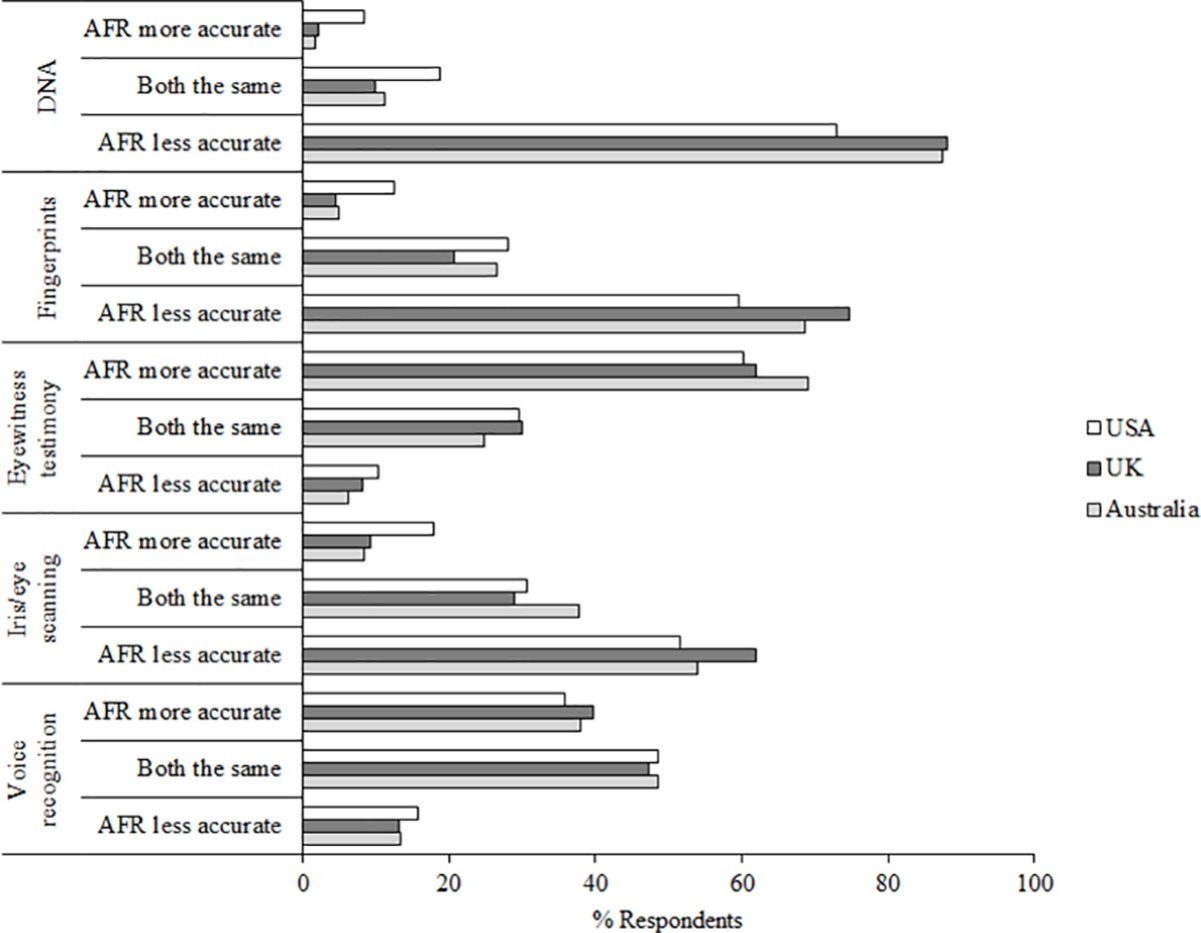
Question 18 data. Responses to question 18 “How accurate do you think this type of facial recognition technology is compared to these different forms of identification?”.

Question 18 asked participants to indicate how accurate they think AFR is compared to other forms of identification. The data are presented in Fig 11.

The majority of participants in all three countries thought that AFR was less accurate than DNA (*M* = 82.81%), fingerprints (*M* = 67.63%) and iris/eye scanning (*M* = 55.83%). In all three of these comparisons, more participants in the USA than both Australia and the UK thought that AFR was more accurate than the other forms of identification. The majority of participants in all countries thought that AFR was more accurate than eyewitness testimony (*M* = 63.71%), and the most common response (but not the majority of participants) in each country was to think that AFR and voice recognition are as accurate as each other (*M* = 48.12%). What may be of note to eyewitness researchers here is that the general public seem to be aware that eyewitness testimony is not always accurate. Also of note is that respondents in the USA seem to see AFR as more in line with DNA and fingerprints as a form of identification. This supports recent research which has shown that both naïve participants and forensic practitioners consider forensic evidence highly reliable, and in fact tend to overestimate its reliability [55].

**Fig 12:**
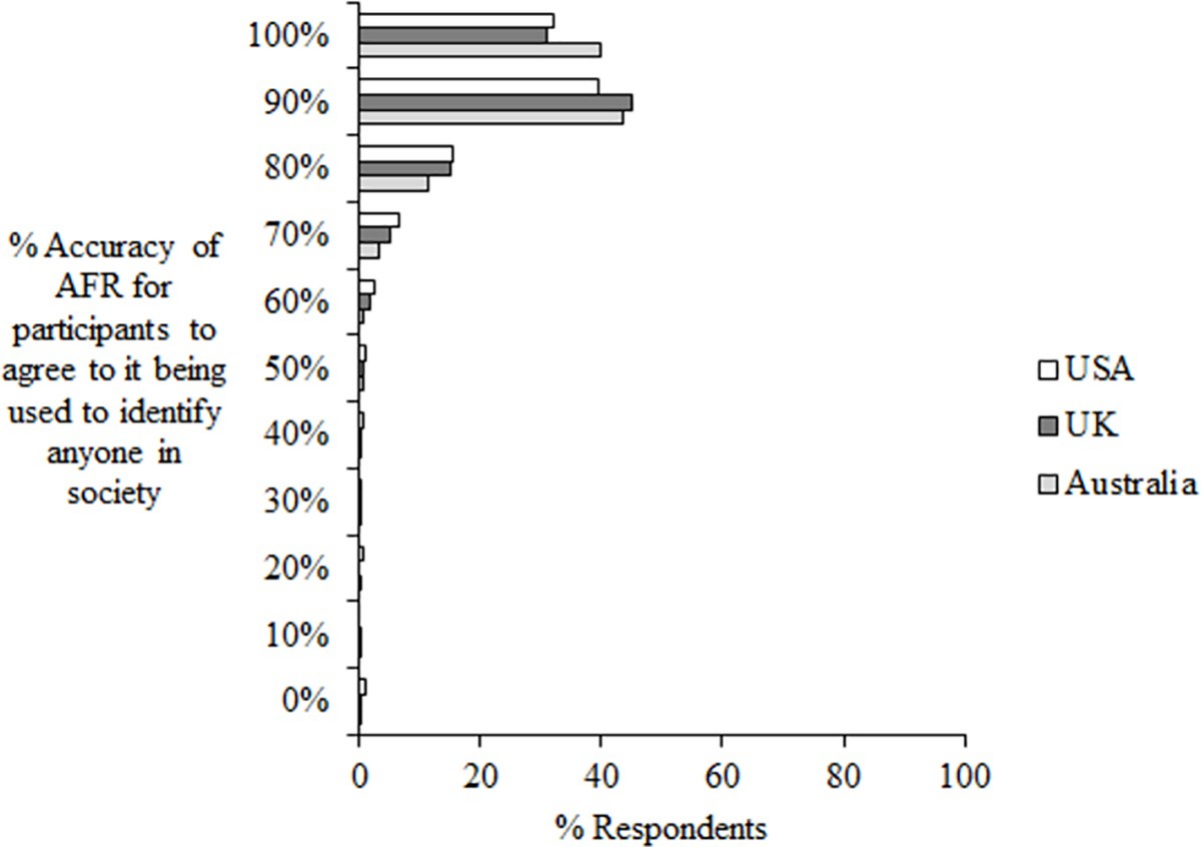
Question 19 data. Percent respondents choosing each percentage interval in response to question 19 “How accurate (in percentage) would this technology need to be in order for you to agree to it being used to identify anyone in society?”.

In response to question 19 which asked “How accurate (in percentage) would this technology need to be in order for you to agree to it being used to identify anyone in society?” (with response choices being given at 10% intervals from 0%-100%), the majority of participants responded 90 or 100% accurate (*M* = 77.00%, see Fig 12).

**Fig 13:**
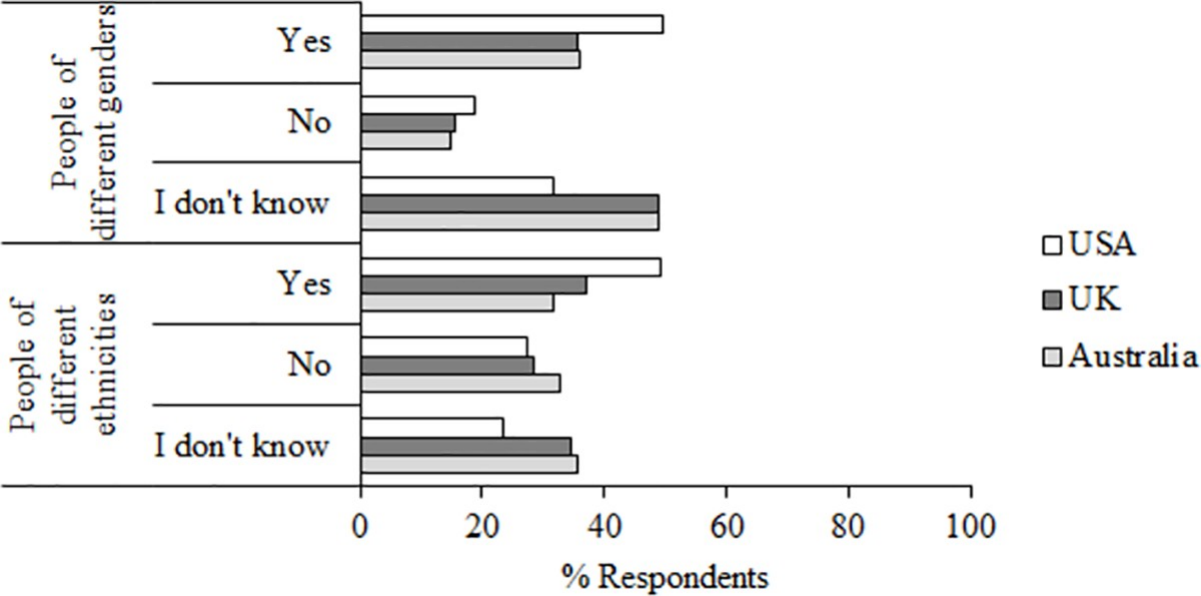
Questions 20–21 data. Percent responses to questions 20–21 “Do you think this technology is equally accurate with people of different genders/ethnicities”.

Questions 20 and 21 asked participants whether they thought that AFR is equally accurate with people of different genders and races (see Fig 13).

Overall, the most common response to the gender question was “I don’t know” (*M* = 43.17%), although more participants in the USA responded “Yes” (*M* = 49.70%) than in either Australia (*M* = 36.06%, z = 6.25, *p* < .001, df = 2,015) or the UK (*M* = 35.59%, z = 6.63, *p* < .001, df, 2,121). For the ethnicity question, the most common response in Australia was “I don’t know” (*M* = 35.56%), but the most common response in the UK was “Yes” (*M* = 37.04%), and this was even more common in the USA (*M* = 49.11%). The relatively high proportions of “I don’t know” responses here seem to reflect uncertainty about the potential limitations of AFR. This tallies with the data from Question 4 which asked “How aware are you of the use and adoption of facial recognition systems in your country?” to which the most common response in each country was “I am aware and I know a little about it”.

**Fig 14:**
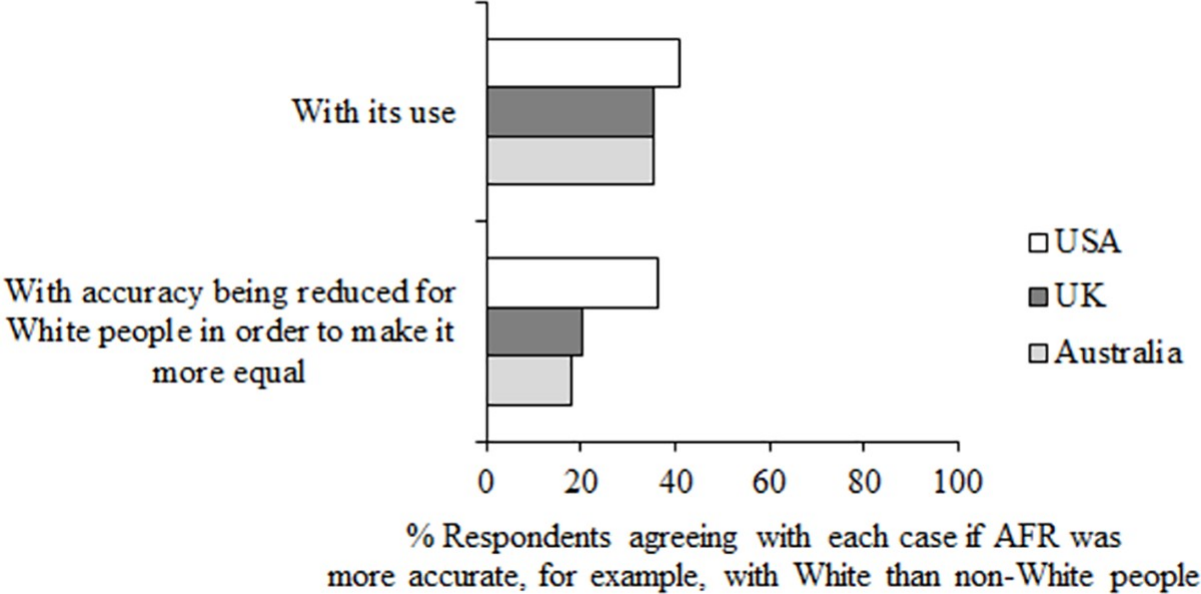
Questions 22–23 data. Percent respondents agreed (responding 4, 5 or 6 on the scale of agreement) with the use of AFR in these circumstances, if it was more accurate with White than non-White people (questions 22 and 23).

Finally, as a thought experiment, questions 22 and 23 asked participants “If this technology was more accurate with, for example, White than non-White people, to what extent do you agree:” both “With its use”, and “With accuracy being reduced for White people in order to make it more equal” (see Fig 14). Having different acceptance rates for different races is a legitimate technical solution to the problem of bias (see [21]), and so it is of interest to find out whether the public would see altering the accuracy of the algorithm for different races as an acceptable solution.

The majority of people in all three countries did not agree with the use of AFR if it was more accurate with White than non-White people (*M* = 37.18%), or with accuracy being reduced for White people in order to make it more equal (*M* = 24.82%). Interestingly, more people in the USA agreed with accuracy being reduced for White people in order to make it more equal (*M* = 41.04%) than in Australia (*M* = 17.98%, z = 11.74, *p* < .001, df = 2,015) or the UK (*M* = 20.14%, z = 10.67, *p* < .001, df = 2,121).

Here we have shown that there is broad agreement between people in Australia, the UK and the USA in their attitudes towards the use of AFR in the criminal justice system, although there are some interesting specific differences whereby people in the UK and Australia tended to have more in common. For example people in the USA were more accepting of the use of AFR to track civilians, less trusting in the police, and more trusting in private companies to use AFR responsibly. Support for the use of AFR depends on both the user and the use case.

## General discussion

Our studies have resulted in four key findings about public attitudes towards the use of AFR in the criminal justice system in different countries:

1. There is a great deal of overlap in public attitudes to AFR in the UK, Australia, and China and the UK, Australia and the USA, but people in the USA and China are generally more positive about AFR than people in the UK and Australia.
2. Public support for the use of AFR depends on the user and the use case. There is more support for police use than private sector use, but even support for the police depends on the use case. Notably, the relative support for particular uses (and users) varied somewhat even between different Western societies (Study 2).
3. Use of AFR, especially in criminal justice settings, appears contingent on accuracy. Respondents expressed concern about relying solely on identification by an algorithm. Use of AFR would seem to require attention to validation for specific uses, extending to demographic classes of individuals.
4. There is some confusion among the public about the accuracy of AFR.

Based on our data, we recommend that developers, system designers, vendors, and users of AFR do more to publicise the use, data privacy, and accuracy of AFR, that it is important for users of AFR to justify their use case and know the capacity of their system, and that governments should provide clear legislation for the use of AFR in criminal justice systems around the world.

The studies presented here have shown that overall, the attitudes of people in the UK, Australia and China (Study 1) and the UK, Australia and the USA (Study 2) are broadly similar when it comes to the use of AFR in criminal justice systems. Some interesting differences between countries did, however, arise at key points. In Study 1 participants in the UK and Australia were more sceptical of the accuracy of AFR, and reported feeling that it was something they see on TV but is not really used in real life. Again participants in the UK and Australia, but not China, had a generally more negative view of AFR and mentioned negative press reports. In addition, participants in the UK and Australia, but again not China, reported being concerned about biases, particularly around the use of AFR with non-White faces, again fuelled by negative press releases.

In Study 2, participants in the USA were more supportive of the use of AFR to track citizens, and to search for people irrespective of whether they had committed a crime than participants in the UK and Australia. Participants in the USA also indicated less trust in the police but more trust in private companies using the technology responsibly than participants in the UK and Australia. Our results are broadly consistent with some other recent surveys of public opinion, but expand on these reports in important ways by asking specific questions about different use cases of AFR, and by comparing public opinion in different countries. To date other surveys have focused exclusively on a single one country [13,15,49,56].

It is worth noting that of course we collected data only from a small subset of people in each country, and that this group of people self-selected to take part. There has been suggestion that data collected from online samples may not generalise to the whole population, even so, results may be useful for indicating the direction if not the magnitude of responses [57]. Other research has suggested that MTurk yields high-quality data [58] and that MTurk responses, specifically regarding security and privacy, are more representative of the population of the USA than responses from a census-representative panel [59]. Therefore although our data may not be representative of the entire population of each country surveyed, we consider extrapolating from this data and making some recommendations based upon it to be an important and worthwhile task.

Our key finding here is that support for the use of AFR depends on the user and the use case. This can be seen clearly in responses to our questions 5–7 in Study 2 (see Fig 6). Support was highest for police use of AFR, then government, then private companies, and within these users, support varied for different use cases. This is consistent with findings from the Ada Lovelace Institute’s review of public perception in the UK which found that 67% of respondents were comfortable (answering 6–10 on a scale of 1–10) with police using AFR to search for suspects in the national police database, whereas only 19% of people were comfortable with the use of AFR in shops to track customers [13]. The relatively high support for police use is also consistent with Australian public attitudes [48] and with a review of Australian’s attitudes towards the use of artificial intelligence in general (not specifically relating to facial recognition) which showed higher support for the use of artificial intelligence in security and justice for public sector users (66.6%) compared to private sector users (60.7%) [56].

In our study, there was higher agreement for the use of AFR to search for people who have committed a crime (police: *M* = 88.86%, government: *M* = 80.42%) and people on a watchlist (*M* = 75.81%), and higher agreement than the Ada Lovelace Institute study [13] for use of AFR by private companies to track people behaving antisocially (*M* = 31.31%). Our results are closer to the results of the London Policing Ethics Panel’s survey of Londoners’ attitudes towards the use of live AFR by police, with 81% agreeing that live AFR should be used to scan crowds at train stations to identify people wanted by the police for serious violent crimes [15]. Interestingly, our results showed that acceptance of private company use of AFR was higher in the USA than in Australia or the UK. This may reflect a more general trust in private industry in the USA than Australia or the UK.

The higher agreement rates in our study and the London Policing Ethics Panel’s survey [15] are likely due to the specificity of the use cases included in the questionnaires. Our study and the London Policing Ethics Panel’s survey [15] were targeted just at AFR use in the criminal justice system, whereas the Ada Lovelace Institute study [13] asked about facial recognition in all sectors including schools and workplaces. We therefore had more space to ask more specific questions in our survey. This is important as it allows us to be clear about public support for different use cases, for example looking at the results of our question 5 (Fig 6, upper panel) asking about police use of AFR, we can see that there is high agreement for use searching for people who have committed a crime, searching for missing persons, and in criminal investigations; but not to track citizens, to search for people irrespective of whether they have committed a crime, in day-to-day policing, or to automate police work. This tallies with the results of our Study 1 ‘intentional positives’ theme in the ‘purpose’ overarching theme where focus group participants spontaneously brought up the use of AFR to search for missing persons as a positive. Our focus group participants, however, also thought that automating police work would be a positive of AFR whereas this was not reflected in the questionnaire.

Interestingly, the reasons people gave to justify their trust, or lack thereof in users of AFR for different use cases was similar here as in other surveys. In our questionnaire (Study 2), among the most common reasons to trust the police, government, and private companies to use AFR responsibly were “It is beneficial for the security of society”, “I trust [the user] to use the technology ethically”, and “The benefits to society outweigh any loss of privacy I might experience”. These sentiments are similar to the two most common views from the London Policing and Ethics Panel’s report [15]: “It will make it easier for the police to catch criminals” and “It makes me feel safer”. The implications of mistakes do not appear to be considered here. The most common reasons people in our questionnaire (Study 2) gave for not trusting the use of AFR were “I am concerned about my data being misused”, “I do not trust that my data will be stored securely”, and “I do not trust [the user] to use the technology ethically”. These support concerns reported in the Ada Lovelace Institute report [13], where 60% of respondents stated “I do not trust them to use the technology ethically”, and in a survey of the public in China which showed that the main reason (over 80%) for concerns about privacy and data security was not knowing who is using the technology [49]. Trust, and issues of data protection were key both in our focus groups (Study 1) and our questionnaire (Study 2).

It is also interesting to note that the Ada Lovelace Institute [13] found that 68% of people were concerned that the use of AFR “normalises surveillance”. Surveillance and the notion of a ‘Big Brother’ state was also frequently mentioned in our focus groups (Study 1) in the ‘privacy’ theme in our overarching theme of ‘society’. From our questionnaire data (Study 2) it is clear that participants did not support the use of AFR for “tracking citizens”, whether used by police, government, or private companies (see Fig 6). The UK’s Data Protection Act 2018 states that the police must only collect biometric data when it is necessary and proportionate, and so tracking citizens and the use of AFR for surveillance of the general population is not something for which AFR is currently used in the UK [4,27]. It is therefore clear that builders, vendors, and users of AFR should all take responsibility for dispelling popular myths such as the surveillance myth by informing the public about the uses of AFR, and the ways in which data are stored and shared.

Data from both of our studies presented here show that there is support from the public for the use of AFR in courts, but reference to the need for other evidence suggests concerns about reliability. This is an important point when considering whether AFR should be admitted, and how it should be presented in court. There is clearly a need for the use of AFR to be regulated across criminal justice systems. The inclusion of a clear framework for police use of AFR in the UK’s Police and Criminal Evidence Act (PACE, 1978) codes of practice would help police forces to make decisions around the use cases in which AFR should be deployed by clarifying what can be seen as necessary and proportionate use. To be clear, it is not the technology which should be legislated for, but the use of the technology, and while general governance frameworks provide a useful basis [60], there appears to be a need for specific legislation for use of AFR in the criminal justice system.

How the various jurisdictional traditions, rules and approaches will apply to AFR is uncertain, though currently unfolding [61]. It seems probable that English courts will be more accommodating than Australian and US courts, following from their acceptance of experts making similarity claims about images. Where AFR and hybrid systems have been formally evaluated and applied to reasonable quality images, as under FVRT, it is likely that similar applications would be admissible in all of these jurisdictions. Issues of design, training, race and bias, as well as validity and reliability, would seem to be issues for the trial and the tier of fact in most jurisdictions. Historically, the British and Americans allowed untested individuals (ie mappers) to testify as facial comparison experts [29,30]. It would be curious if formally evaluated AFR systems, with known levels of performance, were not used to assist with identification. It may be that investigative institutions will work around anticipated risks. Mappers and police specialists may, for example, rely on AFR to generate candidate lists, and then undertake subjective comparisons, in ways that will produce admissible opinions; as least in the UK and US. Though, there may be obligations to explain that nature of the search and the AFR systems relied upon. Because of the exclusion of mappers, Australian courts would seem set to consider the issue of AFR more directly, and presumably AFR designers and emerging police image specialists will be allowed to testify [62].

Our results also showed some public confusion about the accuracy of AFR. 74.09% of participants thought that AFR is accurate at identifying the correct person from a database, dropping to 55.62% who thought AFR is accurate at recognising the same person across changes in appearance (questions 16 & 17, see Fig 10). This shows confusion in that an image captured of a suspect and the image of that person in an existing database will themselves show changes in appearance between the two images. There was also disagreement between participants from different countries on whether AFR is more or less reliable than other types of identification (see Fig 11). This supports recent evidence suggesting that both naïve participants and forensic practitioners consider forensic science evidence highly reliable, and in fact tend to overestimate its reliability [55]. We know from both academic work and standardised testing of algorithms that in the most difficult face recognition situations and with low quality images, algorithms can be variable in their ability to correctly identify people [10], but in ideal conditions most perform very accurately [12]. Therefore it is important that users of AFR know the capacity of their system, for example whether it can be reliably used with low-quality images, and it is vital that the capability of systems is communicated to the public by the builders, vendors and users of AFR.

The capabilities of AFR seem to be decoupled from both public understandings and extant policy. Here we have shown that although there are some specific differences in people’s attitudes towards AFR in different countries, broadly attitudes as well as the reasoning behind them are consistent across the UK, Australia, China and the USA. Support for the use of AFR depends on the user and the use case, and there is only broad support for the use of AFR to secure convictions when it is used in conjunction with other evidence. In addition, there is some confusion around the accuracy of AFR. We recommend a more concerted effort by vendors and users to explain AFR capabilities and use cases to the public, as well as how the data are stored and shared. We also recommend that users of AFR know the capabilities of their system, and that governments legislate the use of AFR in the criminal justice system.

## Supporting information

S1 File Additional information for Study 1. Focus Group Schedule (Study 1).
(DOCX)

S2 File Questionnaire questions (Study 2). Full questions presented to participants in Study 2.
(DOCX)

S3 File Additional information for Study 2. Data Quality/Screening for Questionnaire (Study 2).
(DOCX)

S4 File Australia survey data for Study 2. Australia data.
(XLSX)

S5 File UK survey data for Study 2. UK data.
(XLSX)

S6 File USA survey data for Study 2. USA data.
(XLSX)
