# Supplementary material for: Public attitudes towards the use of automatic facial recognition technology in criminal justice systems around the world
Source: PLoS One. 2021 Oct 13;16(10):e0258241. doi: 10.1371/journal.pone.0258241 (PMC8513835; doi:10.1371/journal.pone.0258241)
Supplement: S1 File — Focus Group Schedule (Study 1). (DOCX) [file pone.0258241.s001.docx]

**S1 File. Additional information for Study 1.** Focus Group Schedule (Study 1)

In Study 1, each focus group was asked the same questions aimed at promoting discussion. Due to the nature of the discussions, some of the questions were covered in prior discussions. Notably, the issue of race was consistently raised before the prompting question concerning race was asked. The schedule of questions was as follows:

1. What do you think facial recognition technology is, and how do you think it works?

Pre question 2. This technology can involve the comparison of an image (eg from CCTV) to a database of images. It can also involve the use of cameras to scan the faces of people passing by. Images can be compared to databases of mugshots (previous arrest images) to identify criminals.

2. How do you feel about this technology being used by police?

in public spaces?

in restricted entry spaces?

in the street?

3. How would you feel about this technology being used if the comparison database didn’t just have photos of wanted criminals, but also had photos of all of us in it?

4. Do you think face recognition technology should be used to investigate any criminal activity? Or do you think this should be limited to certain types of crime?

5. How accurate do you think facial recognition technology is?

6. Do you think this technology is equally accurate with different races of faces?

7. Do you think this technology should be used as evidence in court for the purpose of identification?

8. What are your thoughts on governing this technology – Do you think there should be any laws or policies restricting or promoting the use of this technology?

9. In what situations do you think this technology would be most effective in the future?
