# Supplementary material for: Public attitudes towards the use of automatic facial recognition technology in criminal justice systems around the world
Source: PLoS One. 2021 Oct 13;16(10):e0258241. doi: 10.1371/journal.pone.0258241 (PMC8513835; doi:10.1371/journal.pone.0258241)
Supplement: S2 File — Full questions presented to participants in Study 2. (DOCX) [file pone.0258241.s002.docx]

**S2 File. Questionnaire Questions (Study 2).** Full questions presented to participants in Study 2.

**Section 1. Background knowledge**

Q1. Identity verification is when a device uses either a password or biometrics (personal characteristics like fingerprints) to identify you as the device’s owner. Which of the following methods of identity verification are you aware of? Please select all that apply. [Allow multiple responses]

- Fingerprints
- Iris / eye scanning
- Password
- Voice recognition
- Face recognition / face scanning
- Other (please specify)

Q2. Which of the following methods of identity verification do you currently use? Please select all that apply. [Allow multiple responses]

- Fingerprints
- Iris / eye scanning
- Password
- Voice recognition
- Face recognition / face scanning
- Other (please specify)

Q3. In an ideal world, which of these would you like to rely on for identity verification? Please select all that apply. [Allow multiple responses]

- Fingerprints
- Iris / eye scanning
- Password
- Voice recognition
- Face recognition / face scanning
- Other (please specify)

Facial recognition technology is a biometric system which aims to identify or observe individuals by detecting features associated with a human face. A digital representation of the face is created which can then be compared against a database of stored images. This digital representation may be used to infer characteristics of individuals, and can be matched with similar images to verify a person's identity or uniquely identify individuals.

Q4. How aware are you of the use and adoption of facial recognition systems in your country?

- I am aware and know a lot about it
- I am aware and I know a little about it
- I am aware but I don't know anything about it
- I am not aware of the use and adoption of facial recognition systems at all

**Section 2. Use of facial recognition technology**

Q5. In which of the following ways do you think facial recognition technology is currently being used by the police in your country? Please select all that apply. [Allow multiple responses]

- In their day to day policing
- In criminal investigations
- To search for people who have committed a crime
- To search for people irrespective of whether or not they have committed a crime
- To search for missing persons
- To track citizens
- To automate police work
- I don't know
- Other (please specify)

Q5_1. To Q5_7. [For each of the first 7 options above (Q5)] To what extent do you agree with facial recognition technology being used by the police in your country to [text of option]? Please answer using the scale 1 do not agree at all to 6 strongly agree.

Q6. In which of the following ways do you think facial recognition technology is currently being used by the government in your country? Please select all that apply. [Allow multiple responses]

- To search for people who have committed a crime
- To search for people irrespective of whether or not they have committed a crime
- To search for missing persons
- To track citizens
- To verify identity when accessing government websites
- To prevent fraud when applying for identity documents e.g. passports
- As evidence identifying people in CCTV images in criminal trials
- As evidence identifying people in other digital images (e.g. social media images) in criminal trials
- I don't know
- Other (please specify)

Q6_1. To Q6_8. [For each of the first 8 options above (Q6)] To what extent do you agree with facial recognition technology being used by the government in your country to [text of option]? Please answer using the scale 1 do not agree at all to 6 strongly agree.

Q7. In which of the following ways do you think facial recognition technology is currently being used by private companies in your country? Please select all that apply. [Allow multiple responses]

- To track citizens
- To track people behaving antisocially
- To blacklist people who have previously behaved antisocially
- To share data between businesses e.g. bars and shops in order to blacklist people
- I don't know
- Other (please specify)

Q7_1. To Q7_4. [For each of the first 4 options above (Q6)] To what extent do you agree with facial recognition technology being used by the government in your country to [text of option]? Please answer using the scale 1 do not agree at all to 6 strongly agree.

**Section 3. Trust in the use of facial recognition technology**

Facial recognition technology is used by some police forces as a method of identity verification.  The police may aim to match the digital representations captured by the technology with images present in a database. This database could contain images of every citizen, or only images of individuals on a ‘watchlist’.  Watchlists are created by authorities and contain information about a person of interest, typically those who require close surveillance.  Any images stored for this purpose must be accurate, verifiable and held lawfully by the police.

Q8. How comfortable do you feel with police in your country using facial recognition technology to search for individuals who are on a watchlist? Please answer using the scale 1 not comfortable at all to 6 very comfortable

Q9. How comfortable do you feel with police in your country using facial recognition technology to search for individuals who are not on a watchlist? Please answer using the scale 1 not comfortable at all to 6 very comfortable

Q10. To what extent do you trust the police in your country to use facial recognition technology responsibly? Please answer using the scale 1 I do not trust at all to 6 I trust very much

Q10_1. [Asked only to those responding 4, 5 or 6 to Q10] You said you trust the police to use this technology responsibly, rating your level of trust as [insert rating from Q10]. Which of the following are reasons for your rating? Please select all that apply. [Allow multiple responses]

- I generally trust the police
- It is beneficial for the security of society
- The benefits to society outweigh any loss of privacy I might experience
- It is beneficial for my own personal security
- It does not affect me personally
- I trust that my data will be stored securely
- I trust the police to use the technology ethically
- I can give my consent or opt out
- I am not concerned about my data being misused
- I am not concerned about being tracked by the police
- I don't know
- Other (please specify)

Q10_2. [Asked only to those responding 1, 2 or 3 to Q10] You said you do not trust the police to use this technology responsibly, rating your level of trust as [insert rating from Q10]. Which of the following are reasons for your rating? Please select all that apply. [Allow multiple responses]

- I do not generally trust the police
- It is not beneficial for the security of society
- The loss of privacy I might experience outweighs any benefit to society
- It is not beneficial for my own personal security
- It affects me personally
- I do not trust that my data will be stored securely
- I am concerned about my data being misused
- I do not trust the police to use the technology ethically
- I cannot give my consent or opt out
- I am concerned about being tracked by the police
- It infringes on the privacy of all people in society
- It normalises surveillance
- I don't know
- Other (please specify)

Q11. To what extent do you trust the government in your country to use facial recognition technology responsibly? Please answer using the scale 1 I do not trust at all to 6 I trust very much

Q11_1. [Asked only to those responding 4, 5 or 6 to Q11] You said you trust the government to use this technology responsibly, rating your level of trust as [insert rating from Q11]. Which of the following are reasons for your rating? Please select all that apply. [Allow multiple responses]

- I generally trust the government
- It is beneficial for the security of society
- The benefits to society outweigh any loss of privacy I might experience
- It is beneficial for my own personal security
- It does not affect me personally
- I trust that my data will be stored securely
- I trust the government to use the technology ethically
- I can give my consent or opt out
- I am not concerned about my data being misused
- I am not concerned about being tracked by the government
- I don't know
- Other (please specify)

Q11_2. [Asked only to those responding 1, 2 or 3 to Q11] You said you do not trust the government to use this technology responsibly, rating your level of trust as [insert rating from Q11]. Which of the following are reasons for your rating? Please select all that apply. [Allow multiple responses]

- I do not generally trust the government
- It is not beneficial for the security of society
- The loss of privacy I might experience outweighs any benefit to society
- It is not beneficial for my own personal security
- It affects me personally
- I do not trust that my data will be stored securely
- I am concerned about my data being misused
- I do not trust the government to use the technology ethically
- I cannot give my consent or opt out
- I am concerned about being tracked by the government
- It infringes on the privacy of all people in society
- It normalises surveillance
- I don't know
- Other (please specify)

Q12. To what extent do you trust private companies in your country to use facial recognition technology responsibly? Please answer using the scale 1 I do not trust at all to 6 I trust very much

Q12_1. [Asked only to those responding 4, 5 or 6 to Q12] You said you trust private companies to use this technology responsibly, rating your level of trust as [insert rating from Q12]. Which of the following are reasons for your rating? Please select all that apply. [Allow multiple responses]

- I generally trust private companies
- It is beneficial for the security of society
- The benefits to society outweigh any loss of privacy I might experience
- It is beneficial for my own personal security
- It does not affect me personally
- I trust that my data will be stored securely
- I trust private companies to use the technology ethically
- I can give my consent or opt out
- I am not concerned about my data being misused
- I am not concerned about being tracked by private companies
- I don't know
- Other (please specify)

Q12_2. [Asked only to those responding 1, 2 or 3 to Q12] You said you do not trust private companies to use this technology responsibly, rating your level of trust as [insert rating from Q12]. Which of the following are reasons for your rating? Please select all that apply. [Allow multiple responses]

- I do not generally trust private companies
- It is not beneficial for the security of society
- The loss of privacy I might experience outweighs any benefit to society
- It is not beneficial for my own personal security
- It affects me personally
- I do not trust that my data will be stored securely
- I am concerned about my data being misused
- I do not trust private companies to use the technology ethically
- I cannot give my consent or opt out
- I am concerned about being tracked by private companies
- It infringes on the privacy of all people in society
- It normalises surveillance
- I don't know
- Other (please specify)

**Section 4. The use of facial recognition technology in court**

If facial recognition technology were to be used as evidence in court in your country:

Q13. To what extent do you agree with it being used to secure convictions without other evidence? Please answer using the scale 1 do not agree at all to 6 strongly agree

Q14. To what extent do you agree with it being used to secure convictions in conjunction with other evidence? Please answer using the scale 1 do not agree at all to 6 strongly agree

Q15. To what extent do you agree that it should only be used as a tool to aid investigation and should not be used in court at all? Please answer using the scale 1 do not agree at all to 6 strongly agree

**Section 5. Accuracy of facial recognition technology**

Thinking about facial recognition technology which searches for a target person through databases of images containing multiple different people, please answer the following questions:

Q16. How accurate do you think this technology is at identifying the correct person from a database? Please answer using the scale 1 not accurate at all to 6 very accurate

Q17. How accurate do you think this technology is at recognising the same person across changes in their appearance?

Q18. How accurate do you think this type of facial recognition technology is compared to these different forms of identification? [Options ‘Facial recognition technology is less accurate’, ‘They are both equally accurate’ and ‘Facial recognition technology is more accurate’ presented for the following forms of identification]

- Q18_2. Fingerprints
- Q18_3. Eyewitness testimony
- Q18_4. Iris / eye scanning
- Q18_5. Voice recognition

Q19. How accurate (in percentage) would this technology need to be in order for you to agree to it being used to identify anyone in society?

- 0%
- 10%
- 20%
- 30%
- 40%
- 50%
- 60%
- 70%
- 80%
- 90%
- 100%

Q20. Do you think this technology is equally accurate with people of different genders?

- Yes
- No
- I don't know

Q20_1. [Asked only to those responding 'No' Q20] Which gender(s) do you think this technology is most accurate with? [Allow multiple responses]

- Male
- Female
- Other

Q21. Do you think this technology is equally accurate with people of different ethnicities?

- Yes
- No
- I don't know

Q21_1. [Asked only to those responding 'No' Q21] Which ethnicity(ies) do you think this technology is most accurate with? [Allow multiple responses]

- White
- Asian - Indian
- Asian - Chinese
- Asian - Other
- Black
- Hispanic
- Arab
- Mixed / multiple ethnic groups
- Other (please specify)

Q22. If this technology was more accurate with, for example, white than non-white people, to what extent do you agree with its use? Please answer using the scale 1 do not agree at all to 6 strongly agree.

Q23. If this technology was more accurate with, for example, white than non-white people, to what extent do you agree that accuracy with white people should be reduced in order to make it more equal? Please answer using the scale 1 do not agree at all to 6 strongly agree.
