# Supplementary material for: Public attitudes towards the use of automatic facial recognition technology in criminal justice systems around the world
Source: PLoS One. 2021 Oct 13;16(10):e0258241. doi: 10.1371/journal.pone.0258241 (PMC8513835; doi:10.1371/journal.pone.0258241)
Supplement: S3 File — Data Quality/Screening for Questionnaire (Study 2). (DOCX) [file pone.0258241.s003.docx]

**S3 File. Additional information for Study 2.** Data Quality / Screening for Questionnaire (Study 2)

We included one attention check question and excluded participants who responded incorrectly to it. We also included a captcha (Von Ahn, Blum, Hopper, & Langford, 2003) to screen out bots. Prior to the questionnaire proper, participants completed three English language questions taken from the Cambridge English Scale A2 (basic English) 2004 sample Reading and Writing exam paper (Cambridge Assessment English, 2004). We removed all but the first response from any duplicate IP addresses (available for all countries) which also gave the same demographic information in order to attempt to remove duplicate responses from the same individual.

**References**

Cambridge Assessment English (2004). Cambridge English Scale A2 (basic English) 2004 sample Reading and Writing exam paper. Retrieved from https://www.cambridgeenglish.org/exams-and-tests/key/preparation/

Von Ahn, L., Blum, M., Hopper, N. J., & Langford, J. (2003). CAPTCHA: Using hard AI problems for security. In International conference on the theory and applications of cryptographic techniques (pp. 294-311). Springer, Berlin, Heidelberg.
